# Mutated Toll-like receptor 9 increases Alzheimer’s disease risk by compromising innate immunity protection

**DOI:** 10.1038/s41380-023-02166-0

**Published:** 2023-07-11

**Authors:** Rita Cacace, Lujia Zhou, Elisabeth Hendrickx Van de Craen, Arjan Buist, Julie Hoogmartens, Anne Sieben, Patrick Cras, Rik Vandenberghe, Peter P. De Deyn, Daniel Oehlrich, An De Bondt, Sebastiaan Engelborghs, Diederik Moechars, Christine Van Broeckhoven

**Affiliations:** 1 Neurodegenerative Brain Diseases, VIB Center for Molecular Neurology, VIB Antwerp, Belgium; 2 Department of Biomedical Sciences, University of Antwerp, Antwerp, Belgium; 3 Department of Neuroscience, Janssen Research & Development, a Division of Janssen Pharmaceutica NV, Beerse, Belgium; 4 Department of Neurology, University Hospital Antwerp, Edegem, Belgium; 5 Department of Pathology, University Hospital Antwerp, Edegem, Belgium; 6 Institute Born-Bunge, Antwerp, Belgium; 7 Department of Neurology, University Hospitals Leuven, and Department of Neurosciences, KU Leuven Leuven, Belgium; 8 Department of Neurology and Memory Clinic, Hospital Network Antwerp, Antwerp, Belgium; 9 Discovery Sciences, Janssen Research & Development, a Division of Janssen Pharmaceutica NV, Beerse, Belgium; 10 Department of Neurology, Universitair Ziekenhuis Brussel, and Center for Neurosciences, Vrije Universiteit Brussel, Brussels, Belgium

**Keywords:** Neuroscience, Diseases, Molecular biology

## Abstract

The development of Alzheimer’s disease (AD) involves central and peripheral immune deregulation. Gene identification and studies of AD genetic variants of peripheral immune components may aid understanding of peripheral-central immune crosstalk and facilitate new opportunities for therapeutic intervention. In this study, we have identified in a Flanders-Belgian family a novel variant p.E317D in the Toll-like receptor 9 gene (*TLR9*), co-segregating with EOAD in an autosomal dominant manner. In human, TLR9 is an essential innate and adaptive immune component predominantly expressed in peripheral immune cells. The p.E317D variant caused 50% reduction in TLR9 activation in the NF-κB luciferase assay suggesting that p.E317D is a loss-of-function mutation. Cytokine profiling of human PBMCs upon TLR9 activation revealed a predominantly anti-inflammatory response in contrast to the inflammatory responses from TLR7/8 activation. The cytokines released upon TLR9 activation suppressed inflammation and promoted phagocytosis of Aβ_42_ oligomers in human iPSC-derived microglia. Transcriptome analysis identified upregulation of AXL, RUBICON and associated signaling pathways, which may underline the effects of TLR9 signaling-induced cytokines in regulating the inflammatory status and phagocytic property of microglia. Our data suggest a protective role of TLR9 signaling in AD pathogenesis, and we propose that TLR9 loss-of-function may disrupt a peripheral-central immune crosstalk that promotes dampening of inflammation and clearance of toxic protein species, leading to the build-up of neuroinflammation and pathogenic protein aggregates in AD development.

## Introduction

Alzheimer’s disease (AD) is the most frequent neurodegenerative brain disease [1] affecting over 50 million people worldwide [2]. Most patients present with late-onset AD (LOAD) after the age of 65 years with progressive memory impairment and disturbances in other cognitive functions such as spatial orientation, language, comprehension, reasoning, problem-solving, and judgment [1]. Clinical signs are the result of pathological brain progression over decades resulting in the accumulation of amyloid-β (Aβ) peptides in extracellular plaques surrounded by dystrophic neurites and of intracellular hyperphosphorylated protein tau (p-tau) forming neurofibrillary tangles (NFTs) [3, 4] and the appearance of gliosis and synaptic loss [1]. Apart from LOAD patients, 5–10% of AD patients develop disease between 45–60 years of age [1]. In these early-onset (EO) patients, the disease is mainly genetically determined [1, 2]. In early genetic studies of EOAD families with autosomal dominant disease segregation, three genes were linked to EOAD, i.e., the amyloid precursor protein (*APP*) [5], presenilin 1 (*PSEN1*) [6] and presenilin 2 (*PSEN2*) genes [7]. Only 10% of all EOAD patients [1] can be explained by mutations in one of these 3 causal genes, leaving the majority genetically unexplained. The *ε4* allele of the apolipoprotein E gene (*APOE*) was identified as major risk increasing factor in both LOAD and EOAD patients [1, 2]. Multiple other risk genes and loci were identified by population-based genome-wide association studies (GWAS) [8] and other genetic approaches [2] in LOAD patients, implicating different pathways, besides Aβ processing, in AD etiology, including immune system, synaptic function, lipid metabolism, tau pathway, axonal guidance and cytoskeleton function [2]. Whole genome (WGS) and exome sequencing (WES) techniques facilitated genetic research in EOAD patients [2], discovering rare variants in known risk genes associated to LOAD [9–11] or in novel genes functionally linked to AD pathology [12, 13]. These studies stress the importance of gathering functional data of the effect of rare genetic variants and how these variants can contribute to disease presentation [14].

We identified an EOAD family in Flanders-Belgium in which AD segregates in an autosomal dominant manner, though known mutations in the causal genes were excluded. Follow-up genetic investigation resulted in the discovery of a deleterious missense mutation in the Toll-like receptor 9 (*TLR9*) gene. TLR9 is a crucial player in pathogen elimination and generation of adaptive immune responses [15]. This receptor has been functionally linked to AD and has been a target of multiple in vivo studies [16–22], but genetic mutations in the *TLR9* gene were not identified in AD patients until now. We studied the TLR9 receptor mediated NF-κB activation in a cellular assay and TLR9 physiological activation signaling in human derived peripheral blood mononuclear cells (PBMCs) and assessed the effects of TLR9 signaling induced cytokines on anti-inflammatory and phagocytotic properties of human induced pluripotent stem cells (iPSC)-derived microglia.

## Materials and methods

### Subjects

Diagnosis of AD is based on standard diagnostic criteria of the NINCDS-ADRDA [23] and the National Institute on Aging-Alzheimer’s Association (NIA-AA) [4, 24]. Each AD patient underwent a neuropsychological examination, including Mini-Mental State Examination (MMSE) [25], Montreal Cognitive Assessment (MoCA) [26] and structural neuroimaging, while functional neuroimaging and cerebrospinal fluid analysis was done in a subset of patients [27].

For the control individuals included in the study, subjective memory and neurological or psychiatric antecedents and familial history of neurodegeneration are ruled out via interview Cognitive screening is initially performed using the MMSE (cut-off >25) [25] and later with MoCA (cut-off >25) [26]. Up to 934 unrelated and non-demented individuals were analyzed in this study (mean age at inclusion (AAI), 70.45 ± 9.2 years, range 52–100 years; 58% females).

### Ethical assurances

Written informed consent for participation in clinical genetic and pathological studies was signed by the individual and in case of patients, by the patients or their legal guardian. The study protocols are approved by the Ethics Committee of the University Hospital Antwerp and the University of Antwerp.

### Whole exome sequencing

**Fig. 1:**
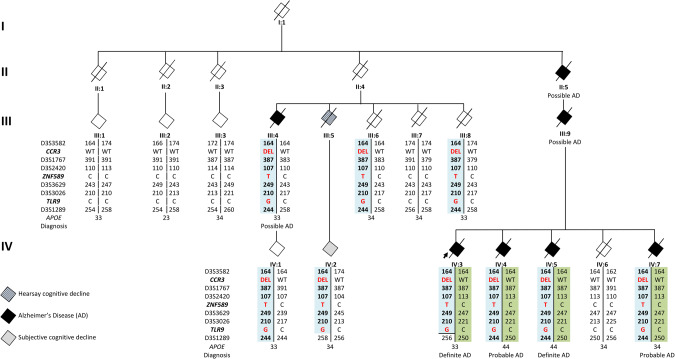
Family pedigree. Blackened symbols represent AD patients, white symbols represent unaffected or at-risk individuals, gray symbol represents an individual with subjective cognitive decline and the striped symbol represents an individual with hearsay cognitive decline. The arrow indicates the proband. The number below the diamonds identifies individuals and on the left side of the pedigree represent generations. Patients IV:3, IV:4, IV:5 and III:4 are included in the WES discovery cohort. DNA was available from generation III (from III:1 to III:4 and from III:5 to III:8) and generation IV (from IV:1 to IV:7) who are depicted in the pedigree and from 7 individuals of generation V, censored from the pedigree as their current age is below the average onset age of the family. STR markers genotypes interspaced by the co-segregating variants and phased haplotypes are reported per individual. Disease haplotype is in light blue. Below the haplotype, *APOE* genotype is reported and clinico(-pathological) diagnosis.

WES data of patients IV:3, IV:4, IV:5 and III:4 (Fig. 1) is generated by AROS Applied Biotechnologies A/S (eurofins Genomics, Denmark), using Nextera Rapid Capture DNA library prep kit (Illumina, CA, USA). WES data of patient IV:7 (Fig. 1) is obtained in the Neuromics Support Facility of the VIB Center for Molecular Neurology, University of Antwerp (Belgium) using SeqCap® EZ Exome v3 kit (Roche, Basel, Switzerland). Burrows-Wheeler Aligner (BWA) [28] is used for sequence alignment to the reference genome GRCh37 (hg19, UCSC Genome Browser). Genome Analysis Toolkit (GATK) Unifed Genotyper [29, 30] is used for variant calling. Data annotation and downstream analysis are performed with the GenomeComb package [31] (http://genomecomb.sourceforge.net/). WES data of the patients except patient IV:7 is analyzed based on autosomal dominant inheritance as outlined in Supplementary Information (SI). Validation and segregation analysis is done using a custom designed massive parallel targeted sequencing (MPS, Agilent Technologies, CA, USA) on a MiSeq® sequencer (Illumina®, San Diego, CA, USA). The same assay in combination with Sanger sequencing (BigDye Terminator Cycle Sequencing kit v3.1; analysis on an ABI 3730 DNA Analyzer, both ThermoFisher Scientific, MA, USA) is used to genotype the Belgian control individuals.

### Chromosome 3 haplotype

The chromosome 3 haplotype is mapped and defined by short tandem repeat (STR) genotyping of 14 STR markers spanning 24.25 cM (Marshfield comprehensive Human genetic maps) [32]. From the Belgian control cohort, DNA of 347 persons is assessed for allelic and haplotype frequency.

### Immunohistochemical examination of brain tissue of patients IV:3 and IV:5

Brain tissue from patients IV:3 and IV:5, carrying *TLR9* p.E317D mutation, are compared with two carriers of *PSEN1* mutations, p.P264L and p.I143T, two patients with sporadic AD (Iba1 staining only) and 2 control persons. Immunostaining is done with anti-CD20 (B-lymphocytes. Clone L26, Roche, Basel Switzerland), anti-CD3 (T-lymphocytes. Clone 2GV6, Roche Basel Switzerland), anti-CD68 (macrophages and microglia. Clone KP1, Agilent Technologies, CA, USA) and anti-Iba1 (microglia, clone EPR16588, ab178846, Abcam, Cambridge, UK) antibodies. The immunohistochemically stained sections of the different brain regions are semi-quantitatively rated (0: no abnormalities; +, ++, +++) by 2 independent blinded neuropathologists (JJM and AS) for CD68, CD20 and CD3 and analyzed by 3 blinded neuropathologists for Iba1 (LM, AM, SA). The evaluation of the microglia was performed based on the recent publication by Schwabenland and colleagues [33].

### TLR9 receptor activation by NF-κB luciferase-based assay for variants modeling

We modeled the p.E317D variant alongside with 3 positive controls, proven to alter TLR9 activation in previous studies: p.W47A, p.F108A [34] and p.P99L [35]. Human *TLR9* full cDNA is cloned behind the CMV promoter into the multiple cloning sites of the pcDNA5/FRT plasmid (ThermoFisher Scientific, MA, USA) and subsequently the NlucP (NanoLuc-PEST) reporter gene behind a minimal promoter with 5 NF-κB response elements, restricted from pNL3.2.NF-κB-RE[NlucP/NF-κB-RE/Hygro] vector (Promega, WI, USA) and cloned into the same TLR9-pcDNA5/FRT plasmid. *TLR9* variants are generated by site-directed mutagenesis (ThermoFisher Scientific, MA, USA). For the TLR9 reporter assay, HEK293 cells are transfected with plasmid expressing both TLR9 variant under a CMV promotor and the destabilized form of NanoLuc luciferase reporter driven by an NF-κB response element (SI). Cells are seeded and stimulated with different concentrations of CpG-ODN or with 0.1 ng/µl TNF-α (Sigma-Aldrich, MO, USA). Fumarizine (Promega, WI, USA) is added 6 h after stimulation and the luminescence is measured in a PerkinElmer EnVision plate reader. Luminescence is calculated relative to the TNF-α response in the same cell line and analyzed with the GraphPad Prism software.

### PBMCs preparation and treatment

Human peripheral blood mononuclear cells (PBMCs) were prepared using ACCUSPINTM System-Histopaque®-1077 (Sigma-Aldrich, MO, USA) following manufacture’s protocol, explained in detail in SI. For treatment, PBMCs were plated and treated with 1 μM TLR9 agonist ODN2216 (InvivoGen, CA, USA), 2.5 μM TLR8 agonist (Janssen, JNJB39224507) or 8.33 μM TLR7 agonist (Janssen, JNJB43025409). Conditioned media were collected 24 h after compound treatment and subjected to Luminex multiplex assays or used for other functional assays. The working concentrations of different agonists were selected based on their individual potencies. A selective TLR9 antagonist (Janssen, JNJB35419342) was used at 500 nM to confirm the specificity of TLR9 agonist in the first pilot experiments.

### Luminex multiplex assay

Profiling of human PBMCs released cytokines were performed using MILLIPLEX MAP Human Cytokine/Chemokine Magnetic Bead Panel I, II, III, IV Plex Immunology Multiplex Assay (Millipore, HCYTMAG-60K-PX38-I-41, HCP2MAG-62K-PX23-II-23, HCYP3MAG-63K-III-11, HCY4MG-64K-PX21-IV-21). In total, 96 human cytokines/chemokines were measured following manufacture’s protocol.

### Microglia differentiation

Human induced pluripotent stem cells (iPSCs) were differentiated to microglia following previously described protocol [36]. Technical description can be found in SI.

### Phagocytosis assays

To measure phagocytosis, human iPSC-derived microglia cells were incubated with 1 μg/mL pHrodo-Aβ_42_ and further incubated with HSC CellMask™ Deep Red stain (ThermoFisher Scientific, MA, USA) before live-cell scanning using Perkin Elmer Opera Phenix (SI). Images were analyzed using Harmony high-content imaging and analysis software (PerkinElmer, version 4.1). Phagocytosis of pHrodo-Aβ_42_ was calculated by dividing total fluorescence intensities of pHrodo by the number of cells quantified from CellMask stain. To assess the effects of individual cytokine or mixture of cytokines induced by TLR9-signaling, microglia cells were pre-treated with 50 ng/ml recombinant IFNα-2a (ProSpec, Israel), IFN-β (PBL Assay Science, NJ, USA), IFN-λ1 (R&D systems, MN, USA), IFN-γ (R&D systems, MN, USA), IL-1RA (ProSpec, Israel), IL-10 (Gibco, ThermoFisher Scientific, MA, USA), SCF (Miltenyi Biotec, Germany), or 10-fold diluted PBMCs conditioned medium for approximately 14 h before phagocytosis assay.

### Induction of IL-1β release from microglia

Induction of IL-1β release in vitro followed previously described protocol [37], detailed in SI. To assess the effects of IFNβ or mixture of cytokines induced by TLR9-signaling on IL-1β release, microglia cells were pre-treated with dilutions of IFNβ, or 10-fold diluted PBMCs conditioned media for approximately 14 h preceding LPS priming.

### RNA extraction and RT-qPCR

RNA extraction was performed using the RNeasy plus mini kit (Qiagen, Germany) following manufacture’s protocol. cDNA synthesis was performed using SuperScript® III (Life Technologies, CA, USA) followed by RT-qPCR using IDT TaqMan assays to detect *AXL* (Hs.PT.56a.1942285), and *RUBCN* (Hs.PT.58.25588809). IDT TaqMan assays for *ACTB* (Hs.PT.39a.22214847) and *TBP* (Hs.PT.58 v.39858774) were used as references. RT-qPCR data were analyzed using Qbase+ software (Biogazelle, Belgium).

### Microarray analysis

RNA extraction was prepared with the RNeasy plus mini kit (Qiagen, Germany) for microarray analysis. Amplification and labeling of total RNA were performed using GeneChip® 3’ IVT Express Kit following manufacture’s protocol (Affymetrix 2004, CA, USA). Biotin-labeled target samples were hybridized to the GeneChip® Clariom_S_Human_HT containing probes for over 18k genes. Target hybridization was processed on the GeneTitan® Instrument according to manufacturer’s instructions provided for Expression Array Plates (P/N 702933). Images were analyzed using the GeneChip® Command Console Software (AGCC) (Affymetrix, CA, USA). Microarray data were processed using the statistical computing R-program (R version 3.4.2) and Bioconductor tools [38]. The gene expression values were normalized using Robust Multi-array Average (RMA) [39]. Individual probes were grouped into gene-specific probe sets based on Entrez Gene using the metadata package clariomshumanhthsentrezg (version 22.0.0) [40].

### Statistics

GraphPad Prism was used for data analysis. One-way ANOVA with Dunnett’s multiple comparisons or Fisher’s LDS pairwise comparisons were used as indicated. Values are mean ± SD, numbers (*n*) are indicated in figures. ∗*p* < 0.05, ∗∗*p* < 0.01, ∗∗∗*p* < 0.001, ∗∗∗∗*p* < 0.0001, and ns (not significant).

## Results

### Belgian AD family

**Fig. 2:**
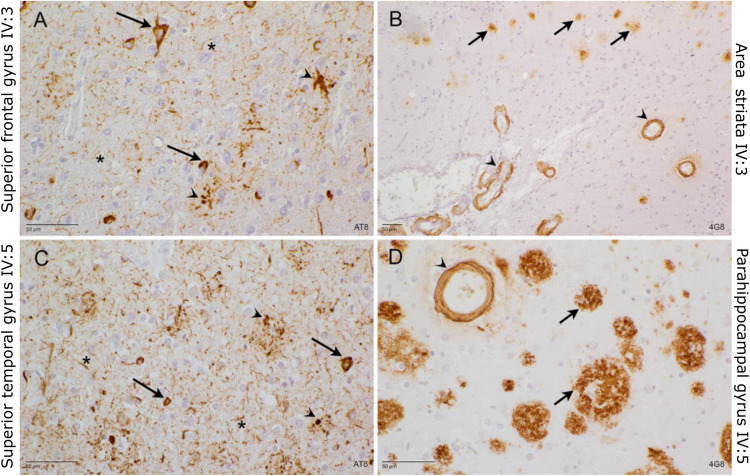
Neuropathological examination of brain tissue of IV:3 and VI:5. **A** Superior frontal gyrus of IV:3: AT8 stain. Many neurofibrillary tangles (arrow), as well as dystrophic neurites (arrowhead) and neuritic threads (asterisk) are detected. **B** Area striata of IV3: 4G8 stain. Small magnification to show the abundance of cerebral amyloid angiopathy (CAA, arrowhead) and senile plaques (arrow). **C** Superior temporal gyrus of IV:5 AT8 stain. Many neuritic threads (asterisk) and dystrophic neurites (arrowhead) affect the temporal cortex. Many neurons contain neurofibrillary tangles (arrow). **D** Parahippocampal gyrus of IV:5: 4G8 stain. Many large senile plaques (arrow), again in the presence of cerebral amyloid angiopathy (arrowhead).

From the family, we selected all individuals with DNA and/or clinical data (Fig. 1). DNA of 7 family members from generation III and 7 from IV was available (Fig. 1, SI Tables 1 and 2); family members of generations I and II are deceased and clinical information was scarce. Of generation V, we obtained DNA of 7 family members, but this generation is not shown in Fig. 1 to protect the privacy of this offspring who is young and asymptomatic. The genetic information of generation V was used to define and phase the carrier’s disease haplotype. The family proband IV:3 was diagnosed with EOAD at the age of 52 years (Fig. 1). In the branch from II:5, besides patient IV:3 (definite AD), there are three additional affected siblings, IV:4 (probable AD), IV:5 (definite AD) and IV:7 (probable AD), as well as one parent (III:9) and one grandparent (II:5) both with possible AD diagnosis. The inheritance pattern is indicative of autosomal dominant inheritance. Detailed clinical information of the patients is reported in SI Table 1. Neuropathological examination of post-mortem brain tissue of patients IV:3 and IV:5, showed severe AD neuropathology and cerebral amyloid angiopathy (CAA) in both patients (Fig. 2). In the branch of II:4 (Fig. 1), patient III:4 was diagnosed with possible AD. Individual IV:1, offspring of patient III:4, asymptomatic, was 63 years at last examination, which is still in the onset age range of the family, for this person no clinical examination was performed. Patient III:5 did not undergo an in-depth neurological examination, but a cognitive decline five years prior death was mentioned by first degree relatives. Cognitive decline was reported for patient IV:2, offspring of patient III:5, but a clear diagnosis was not formulated. Family member III:6 suffered from a subarachnoid bleeding (SAB) and 2 ischemic cerebrovascular accidents (iCVA). Cognitive decline was not reported, but MRI, showed cortico-subcortical atrophy. No clinical data is retrieved for individual III:8. All healthy and at-risk individuals are reported in SI Table 2. The average onset age in the family is 57.8 years (range 58–64). From generation V, the younger family members are on average 44.7 years (range 39–51), which is below the average onset age of the patients in the family and, amongst them, the carriers of the *TLR9* mutation are still asymptomatic.

### Genetic screening of known genes

We screened patients IV:3, IV:4 for mutations in *APP*, including duplications in the *APP* locus, and in *PSEN1*, *PSEN2*, *MAPT*, *GRN*, *TARDBP*, but no pathogenic mutations were identified. The *C9orf72* repeat expansion analysis did not identify expanded repeat alleles [41]. Premature termination codon (PTC) mutations were not identified in *ABCA7* in patients IV:3, IV:4, III:4, IV:5, IV:7. Also, patients IV:3 and IV:4, were screened for the *ABCA7* variable number tandem repeat (VNTR) by Southern blot as previously described [42], in both patients the VNTR length was in the normal range (<5720 bp) [42]. No PTC mutations were identified in *SORL1* in patients IV:3, IV:4, III:4, IV:5, IV:7. Patient IV:3 tested negative also in a previous study [43]. There were no risk variants in *TREM2* in IV:3, IV:4, III:4, IV:5, IV:7. The patient IV:3, was initially included in the study of Cuyvers et al., 2014 [44]. A polygenic risk score (GRS) was calculated for patient IV:4 who was included in a previous study [45]. This IV:4 patient has an *APOE* ε4ε4 genotype, a positive family history and early onset age and scored in the high-risk category g5 (ALL_WA model wGRS = 4.96) [45], where a great amount of discriminative ability of the GRS was attributable to the *APOE* genotype, positive family history of AD and early onset age. In the AD family, that we are screening in the project of this paper, the *APOE* genotypes are not directly accounting for the disease, with patient IV:3 presenting an *APOE* ε3ε3 genotype. Taken all the genetic screening results we considered that this family is genetically unresolved.

### WES data of the Belgian AD family

We started our quest to identify the genetic role in the family by WES screening of the patients IV:3, IV:4, IV:5 and III:4, Fig. 1 and SI Table 1) selected as discovery cohort. The WES data of the 4 patients had an average 20× target coverage of 84.93% (range 78.5% in IV:3 to 91.8% in III:4), reaching 91.65% covered at least 10×. The 4 exomes shared 87221 variants of which 27375 variants called as heterozygous in all 4 patients. Filtering the WES data for quality, frequency, and impact on protein retained 10 coding variants. Validation of the variants and co-segregation in the family resulted in three variants located on chromosome 3 and co-segregating with AD: *CCR3* p.F249Hfs*23 (rs561062190), *ZNF589* p.T355M (rs376706270) and *TLR9* p.E317D (novel) (Fig. 1 and SI Table 3). Haplotype sharing, using STR markers, defined a haplotype delimited by D3S3727 and D3S1289 (15.34 cM; 23.8 Mb) on chr3p24.1-p14.3 (SI Fig. 1, SI Table 4) across the three variants. Direct genotyping in a Belgian cohort of controls identified 3 controls carrying the *CCR3* p.F249Hfs*23 (3/934, 0.32%), one (DR1578) also carrying the *ZNF589* p.T355M variant (1/934, 0.11%). STR genotyping of DR1578 showed the same haplotype as in the family though with a recombination between the *ZNF589* and the D3S3629 STR (SI Fig. 1 and SI Table 5), excluding *TLR9* from the shared haplotype of the control person. We observed that *CCR3* c.738delGT (p.F249Hfs*23), located in the last gene exon, leads to NMD escape of the mutated transcript (SI Fig. 2). Both *ZNF589*, p.T355M and *CCR3* c.738delGT (p.F249Hfs*23), are ultra-rare in the gnomAD database [46], while *TLR9* p.E317D is a novel variant. All three variants are absent from the HEX database of 478 neuropathological healthy controls with an age at inclusion over 60 years (https://www.alzforum.org/exomes/hex) (SI Table 3).

### Brains of *TLR9* p.E317D carriers show alterations of microglia morphology

Brain tissue of two *TLR9* p.E317D carrier patients (IV:3 and IV:5), two *PSEN1* carriers (p.P264L and p.I143T) and two neurologically healthy controls were examined for CD20, CD3 and CD68. No infiltration of lymphocyte T and B was detected in all areas. In all patients and controls, sparse macrophages and microglia (CD68 positive) were found throughout the white matter. In the control brains there was no CD68 immunoreactivity in the cortex in comparison with the *TLR9* (p.E317D) brains and the *PSEN1* (p.P264L and p.I143T) brains. In both *TLR9* and *PSEN1* carriers considerable CD68 immunoreactivity was observed in the cortex, indicating neuroinflammation likely in response to tissue damage [47] (SI Figs. 3–4). While observed at relatively young age, there was no difference in the severity of the CD68 immunoreactivity between the *PSEN1* and *TLR9* carriers (SI Table 6) upon semi-quantitative analysis. Notably, in the neuropathology CAA was detected in the *TLR9* p.E317D carriers IV:3 and IV:5 (Fig. 2).

**Fig. 3:**
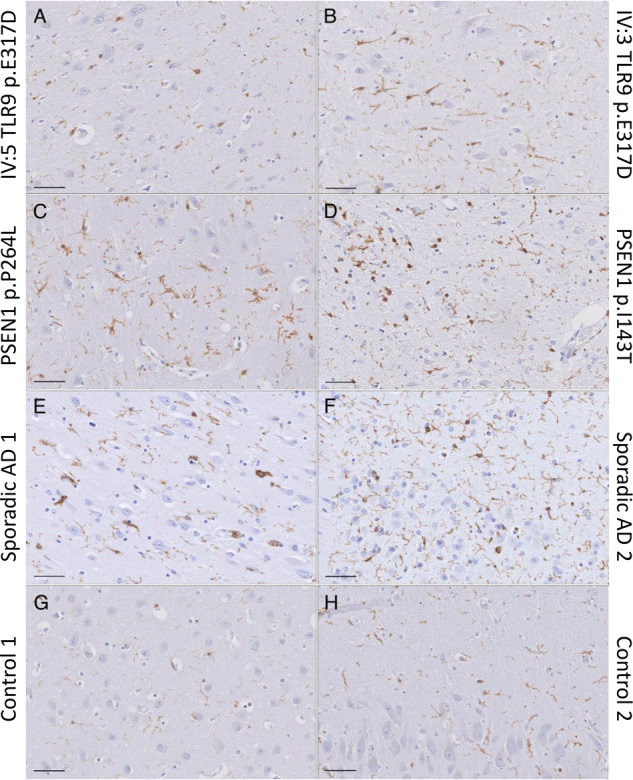
Iba1 staining of hippocampal cortex. Scale bar 50 μm. **A** IV:5 and (**B**) IV:3; both patients carry the TRL9 p.E317D variant, (**C**) and (**D**) are AD patients with *PSEN*1 p.P264L and p.I143T respectively, (**E**) and (**F**) are two sporadic AD patients and (**G**) and (**H**) are neurologically healthy controls. The TLR9 p.E317D mutation carriers (Fig. 3A, B) and the other AD (**C**–**F**) showed alterations of microglia morphology. In the TLR9 p.E317D (**A**, **B**) the microglia cells appeared more condensed with less processes and ramifications and with the least overlap in microglia territory.

The Iba1 staining (Fig. 3 and SI Fig. 5), allowed the blinded raters to separate the TLR9 p.E317D mutation carriers and other AD cases (i.e., *PSEN1* mutation carriers and sporadic AD patients) from the neurologically healthy control individuals. Hippocampal cortex (Fig. 3) and frontal cortex (SI Fig. 5) are shown. The neurologically healthy control individuals presented with the least amount of Iba1 immunoreactive microglia (Fig. 3G, H). The morphology of the microglia was compatible with not activated status [33] and the microglia were evenly distributed throughout the cortex. The TLR9 p.E317D mutation carriers (Fig. 3A, B) and the other AD cases [*PSEN1* mutation carriers (Fig. 3C, D) and sporadic AD (Fig. 3E, F)] showed alterations of microglia morphology. In the TLR9 p.E317D (Fig. 3A, B) the microglia cells appeared more condensed with less processes and ramification.

### TLR9 receptor activation assay for variant p.E317D modeling

**Fig. 4:**
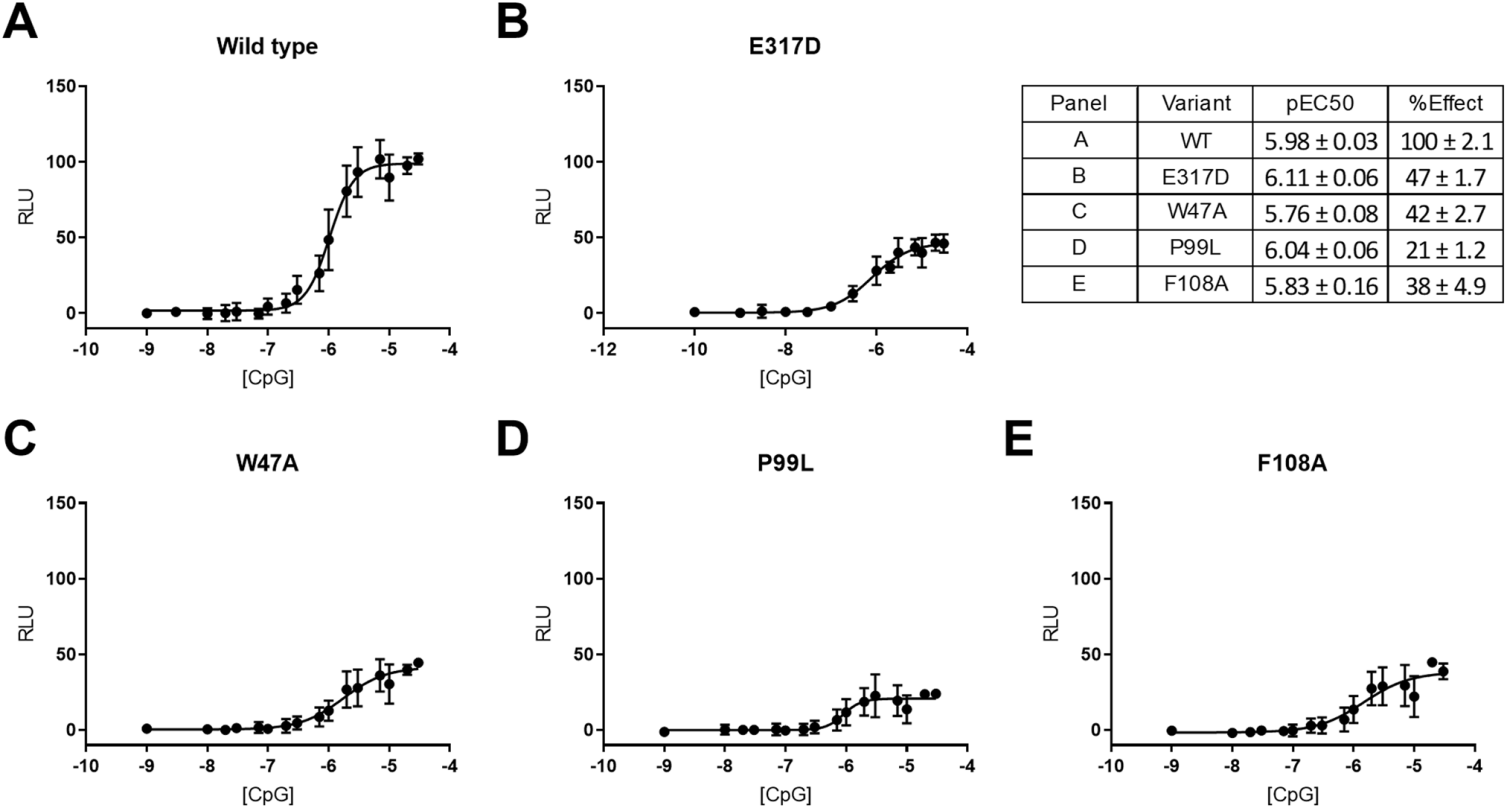
Effect of TLR9 p.E317D variant on signaling. HEK293 cells transiently transfected with a plasmid containing TLR9 wild type (**A**) or mutated (**B**–**E**) and a destabilized form of NanoLuc luciferase reporter driven by an NF-κB response element. TLR9 activation was achieved with different concentrations of the CpG ODN2006 or 0.1 ng/µl TNF-α as control for expression levels. Luminescence is expressed relative to the TNF-α response in the same cell line with the TLR9 wild type response set at 100%. The average of 3 independent experiments is shown. In the table, logarithmic scale of the half maximal effective concentration (pEC50) and maximum responses (% Effect) are reported.

TLR9 is a single pass transmembrane protein which is localized in the endosomal compartment and is involved in the recognition of pathogens nucleic acids such as unmethylated CpG-ODN. The co-segregating variant p.E317D is positioned in a conserved amino acid residue within the CpG-ODN binding pocket of TLR9 involved in DNA sensing [34, 48] and predicted to be deleterious with a Combined Annotation Dependent Depletion (CADD) [49] score of 22.4. The mutation does not affect transcript or protein expression (SI Fig. 6) in lymphoblastoid cell lines (LCL). We demonstrate that p.E317D causes a reduction of TLR9 activation (effect (%) 47 ± 1.7; Fig. 4B and SI Fig. 7). This effect was like the positive control variants p.W47A (Fig. 4C), p.F108A [34] and p.P99L [35] (Fig. 4D, E respectively), known to alter receptor response. The half maximal effective concentration (EC50) remained unaltered in the modeled variants, independently on the direction of the effect (Fig. 4).

### Profiling of cytokines induced by TLR9-signaling

In human, TLR9 expression is mostly detected in peripheral immune cells [50] but is not detected in brain tissue-derived microglia cells (source: https://www.brainrnaseq.org/) [51, 52] nor in human iPSC-derived microglia (SI Fig. 8). Thus, we interrogated whether TLR9 could be involved in AD pathogenesis by regulating peripheral immune response. We performed cytokine profiling of human PBMCs upon TLR9 activation, in comparison with the responses of TLR7 and TLR8 activation. Among the 96 target analytes, TLR9, TLR7, and TLR8 activation induced 22, 34 and 36 cytokines, respectively (SI Fig. 9A–C). First, we observed that TLR7 and TLR8 induced similar levels of pro-inflammatory cytokines including IL-1β, IL-1α, IL-12p40, TNFα, IFN-γ, IL-6, all of which were hundred-to-thousand-fold higher than those induced by TLR9 (SI Fig. 9D). Second, TLR7, 8 and 9 induced similar levels of anti-inflammatory cytokine IL-1RA while TLR9 induced IFN-β (SI Fig. 9D), an important mediator for anti-inflammatory responses and an antagonist for IL-1 and IFN-γ pro-inflammatory signaling [53–55]. Cytokine profiling upon TLR9 activation was repeated in human PBMCs from five additional healthy donors and the profiles were consistent among donors (SI Fig. 10).

### TLR9-signaling induced cytokines by PBMCs reduce IL-1β and TNFα release from human iPSC-derived microglia

**Fig. 5:**
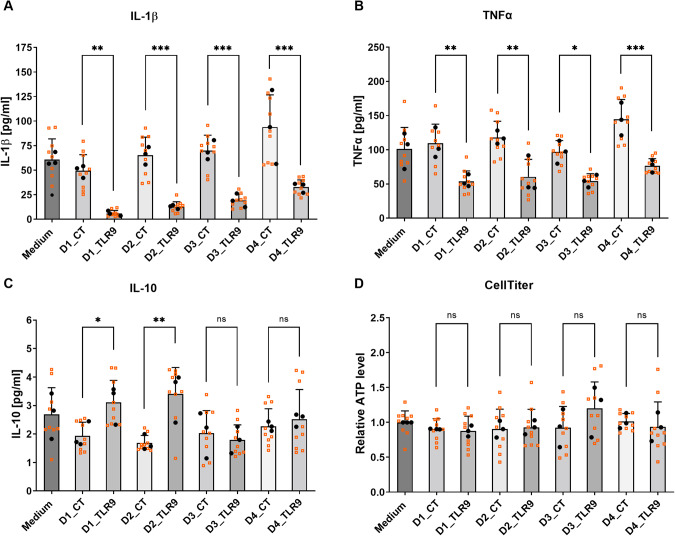
TLR9-signaling induced cytokines reduce IL-1β and TNFα release from human iPSC-derived microglia. Conditioned media were collected from PBMCs after treatment with 1 μM TLR9 agonist ODN2216 (Donor#_TLR9; containing mixture of TLR9-signaling induced cytokines) or vehicle (Donor#_CT) and were diluted 10-fold to pre-treat human iPSC-derived microglial cultures for ~14 h. After the pre-treatment, microglial cultures were subjected to LPS priming for 4 h and nigericin stimulation (inflammasome activation) for 2 h. The supernatants from microglial cultures were collected for cytokine measurement by MSD multiplex assay. The concentrations of measured cytokines are plotted in chart (**A**) IL-1β, (**B**) TNFα, (**C**) IL-10, and the CellTiter measurements are plotted in chart (**D**). PBMCs were prepared from four healthy donors (Donor 1-Donor 4). Biological replicates from *n* = 3 independent experiments (each experiment with 3 biological replicates) are plotted in small-sized orange squares, mean values of biological replicates per experiment are plotted in black dots. Mean ± SD, One-way ANOVA with Fisher’s LDS pairwise comparisons of mean values from independent experiments. ∗*p* < 0.05, ∗∗*p* < 0.01, ∗∗∗*p* < 0.001, and ns (not significant).

IFN-β, previously reported to prevent IL-1 production [37], was the major anti-inflammatory cytokine induced by TLR9-signaling. To confirm the effects of IFN-β on IL-1 production, we pre-treated human iPSC-derived microglial cultures with recombinant IFN-β and induced IL-1β release by LPS-priming plus nigericin-mediated inflammasome activation. Pre-treatment of the microglia with IFN-β reduced IL-1β release in a dose-dependent manner as expected (SI Fig. 11). We asked whether the IFN-β within the cytokine pool released by PBMCs in response to TLR9 activation, could exert anti-inflammatory by blocking IL-1 production and applied conditioned media of human PBMCs, with or without TLR9 activation, to human microglial cultures preceding LPS-priming and nigericin-stimulation. PBMCs conditioned media containing TLR9-signaling induced cytokines, reduced IL-1β release (Fig. 5A). Such media also reduced release of TNFα (Fig. 5B), another key pro-inflammatory factor implicated in neuroinflammation. The observed effects on IL-1β and TNFα were consistent for PBMCs samples prepared from four healthy donors. In contrast, PBMCs conditioned media from different donors did not show consistent effects on the levels of IL-10 (Fig. 5C). ATP measurement showed that treatment of PBMCs conditioned media with or without TLR9-induced cytokines did not affect microglial cell viability (Fig. 5D).

### TLR9 signaling induced cytokines by PBMCs increase phagocytosis of Aβ_42_ in human iPSC-derived microglia

**Fig. 6:**
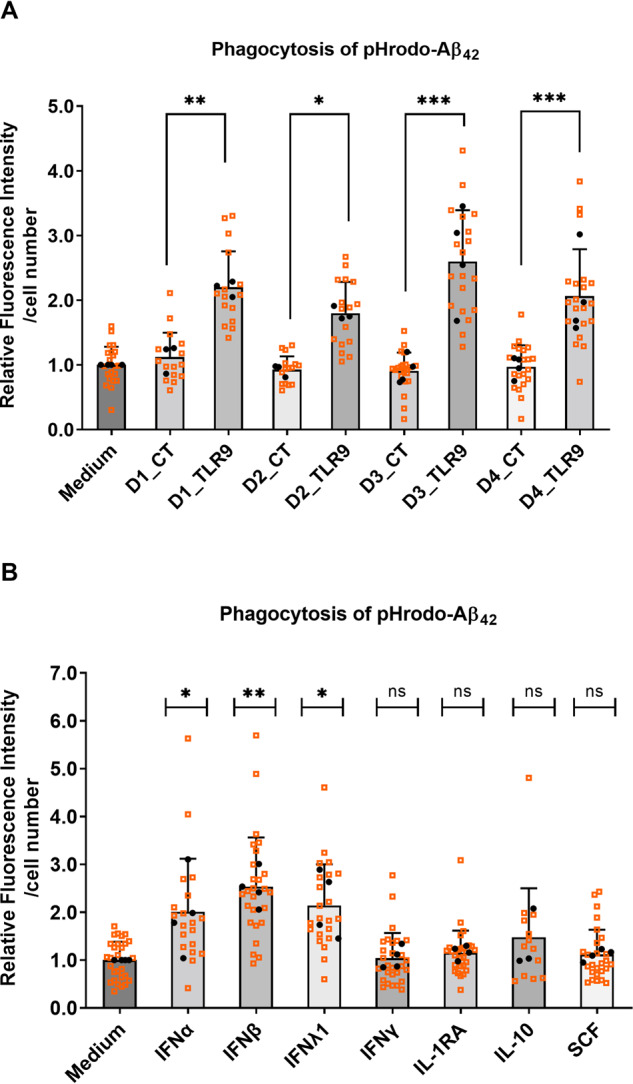
TLR9-signaling induced cytokines increase phagocytosis of pHrodo-labeled Aβ_42_ in human iPSC-derived microglia. Conditioned media were collected from PBMCs after treatment with 1 μM TLR9 agonist ODN2216 (Donor#_TLR9; containing mixture of TLR9-signaling induced cytokines) or vehicle (Donor#_CT) and were diluted 10-fold to pre-treat human iPSC-derived microglial cultures for ~14 h. After the pre-treatment, microglial cultures were incubated with pHrodo-labeled Aβ_42_ (1 μg/mL) for 4 h before live-cell scanning with the presence of CellMask™ deep red stain. Phagocytosis of pHrodo-Aβ_42_ were quantified by dividing total fluorescence intensities of pHrodo by total cell numbers (**A**). Alternatively, recombinant cytokines (IFNα-2a, IFN-β, IFN-λ1, IFN-γ, IL-1RA, IL-10, SCF) at concentration of 50 ng/ mL were used to pre-treat microglial cultures preceding phagocytosis assay, phagocytosis of pHrodo-Aβ_42_ were quantified accordingly (**B**). Biological replicates from *n* = 3 or 4 experiments (each experiment with 6 biological replicates) are plotted in small-sized orange squares, mean values of biological replicates per experiment were plotted in black dots. Mean ± SD, One-way ANOVA with Fisher’s LDS pairwise comparisons (**A**) or Dunnett’s multiple comparisons (**B**) of mean values from independent experiments. ∗*p* < 0.05, ∗∗*p* < 0.01, ∗∗∗*p* < 0.001, and ns (not significant).

Cytokines are a class of signaling proteins known to be involved in regulating phagocytic properties of phagocytes [56]. PBMCs conditioned media containing TLR9-signaling released cytokines increased microglial phagocytosis of pHrodo-labeled Aβ_42_ (Fig. 6A). This observation was consistent for PBMCs samples prepared from four donors.

To identify the functional components from PBMCs conditioned media that affect phagocytosis, we assessed a set of TLR9-signaling induced cytokines individually using recombinant proteins. Among the tested cytokines, IFN-α, IFN-β and IFN-λ at 50 ng/mL increased microglial phagocytosis of pHrodo-labeled Aβ_42_, whereas IFN-γ, IL-1RA, IL-10 and SCF at the same concentration had no significant effects on phagocytosis (Fig. 6B). Of note, when using Phrodo-Zymosan bioparticles, the IFNs stimulation suppressed the phagocytosis (SI Fig. 12) confirming previous data [57] and highlighting the different phagocytic responses of the microglia when using pHrodo-labeled Aβ_42_ as AD relevant protein. To explore the mechanisms underlying the effects of IFNs on phagocytosis, we performed microarray transcriptome analysis of microglial cells treated with different IFNs at a high (50 ng/mL) and a low concentration (50 pg/mL). At 50 ng/mL, IFN-α, β, λ and γ induced differential expression of 2378, 2506, 252 and 1194 genes, respectively (SI Fig. 13; SI excel file); at 50 pg/mL, IFN-α, λ and γ induced much lower numbers of differentially expressed genes (248, 0 and 360, respectively), whereas IFN-β still induced a high number of genes (1415). This suggests that compared to IFN-α, λ and γ, IFN-β has relatively higher affinity to its receptor in microglia, predicting its predominant role when in a mixture with other IFNs.

To dissect how IFN-α, β and λ increased phagocytosis, we performed a principal component analysis (PCA) of the microarray transcriptome data focusing on 212 genes involved in phagocytosis pathways. As shown in the spectral map (SI Fig. 14), a set of genes including *STAP-1*, *AXL* and *KIAA0226* (*RUBICON*) drives the differences between IFN-α, β and λ treated samples with the control samples along the PC1 axis. Among those genes, *AXL* and *RUBICON* are described essential regulators of phagocytosis [58, 59].

We further questioned whether TLR9-sigaling induced cytokines could indeed affect the phagocytosis genes predicted using recombinant IFNs. We treated microglial cultures with PBMCs conditioned media and analyzed gene expression by RT-qPCR. PBMCs conditioned media containing TLR9-sigaling induced cytokines, increased *AXL* and *RUBICON* mRNA expression approximately 17- and 5- fold, respectively (SI Fig. 15).

## Discussion

We studied a Flanders-Belgian EOAD family presenting with autosomal dominant inheritance without pathogenic mutations or high-risk penetrant variants in known genes. Using WES we discovered for the first time a mutation in *TLR9* co-segregating with AD. The penetrance of *TLR9* p.E317D cannot be fully assessed as in the family there are *TLR9* p.E317D carriers that are younger than the average onset age of disease (i.e., IV:1) or for whom a formal clinical diagnosis could not be yet formulated (i.e., IV:2) or else, presented with co-morbidities that made it difficult to establish a clinical neurological diagnosis (i.e., III:6 with SAB and iCVA). It is known that, modulation of TLR9 function, via CpG ODN agonists, showed amelioration of CAA levels, cortical amyloid burden in association with behavioral improvements in animal models of AD [16–19, 22], with a non-negligible safety profile in non-human primate model of sporadic AD pathology [22].

In humans, TLR9 is primarily expressed in peripheral immune cells including plasmacytoid dendritic cells (pDC) and B cells [60]; while in mice, it is expressed also in microglia [60]. There is yet no consensus on the TLR9 protein expression in human brain [61], but there is indication of post transcriptional regulation of TLR9 in mice [62] complicating the interpretation of RNA-based expression studies. Available RNA sequencing databases, like the Brain RNA-Seq from the Barres lab (https://www.brainrnaseq.org/) [51, 52] show TLR9 expression being abundant across microglia and macrophages in rodents while being not expressed in the human counterpart, including, as we show, human iPSC-derived microglia. For this reason, TLR9 p.E317D mutation is not expected to directly impact microglia response to triggers. The differential TLR9 expression pattern between human and mice is known to affects the validity and the translational potential of studies from mice to human [19]. Consequently, we investigated TLR9 function in human derived PBMC and developed a hypothesis on how peripheral TLR9 activity could be involved in AD pathogenesis. Increasing evidence suggests that peripheral immune responses and systemic inflammatory cytokines affect the central nervous system (CNS) homeostasis [63–67]. We asked whether TLR9 is involved in AD pathogenesis by regulating the peripheral immune response. First, we show that the cytokine profiling of human PBMCs upon TLR9 activation present a predominant anti-inflammatory response, including an exclusive induction of IFN-β compared with TLR7/8 activation. IFN-β is reported to prevent the production of IL-1 [37], a master regulator of inflammatory reactions in immune system and a key pro-inflammatory factor implicated in neuroinflammation. Secondly, we demonstrated that IFN-β reduced the release of IL-1β from iPSC-derived microglia, providing the link between the peripheral activation of TLR9 and a CNS effect. We confirmed this link by showing that conditioned media from PBMCs containing TLR9-signaling induced cytokines reduced release of IL-1β as well as release of TNFα, another key pro-inflammatory factor implicated in neuroinflammation. Additionally, the conditioned media increased the microglial phagocytosis of Aβ_42_ with AXL and RUBICON as key regulators of phagocytosis [58, 59], recently implicated in AD pathophysiology [68, 69]. Failure of the immune system to clear Aβ, rather than an overproduction of the Aβ peptides, is likely the disease mechanism involving TLR9. It is expected that if innate immune cells have an impaired ability to limit Aβ accumulation they could switch into a pathological state with detrimental effect [70]. Unfortunately, we could not perform the set of experiments, described in this study, in patient derived LCL with *TLR9* p.E317D mutation. The PBMCs transformed into LCL using Epstein Barr virus caused the expression of viral latent membrane protein 1 (LMP1), which is a known negative regulator of TLR9 activation [71], preventing us from delivering a direct link between the identified mutation and the proposed disease mechanism. Furthermore, no significant differences in TLR9 mRNA and protein expression are observed in LCL between p.E317D carriers and non-carriers, indicating that the mutation is not affecting expression but is having an effect on the protein signaling, as we show in the luciferase-based assay. A relevant aspect to consider is the stoichiometry of TLR9, which works as protein dimer, with the mutated allele potentially acting as dominant negative and affecting the wild-type allele function.

A recent work [72] supports a model where neuroimmune signaling in tauopathies, including AD, converge on viral response pathways, suggesting an important causal connection between viral defense and pathological tau protein [72]. The molecular machineries governing inflammatory response are conserved between microglia and peripheral macrophage. Previous studies have shown that IFN-β suppresses IL-1 production in macrophage [37, 73]. We consider that the currently identified molecular mechanism for TLR9-signaling promoting anti-inflammation is not limited to microglia in the CNS, but rather predicts a similar anti-inflammatory effect on macrophage in the periphery. Emerging clinical evidence suggests that systemic inflammation is associated with longitudinal changes in cognitive performance [20, 74, 75], supporting the crosstalk between systemic inflammation and CNS homeostasis and functionality.

In conclusion, our family-based genetic study identified a *TLR9* mutation co-segregating with EOAD and altering the microglia activation and morphology in the brain of the patients carriers of p.E317D and negatively affecting receptor signaling. A role for TLR9 in neuroinflammation has been supported by several in vivo studies in AD [16–22]. Here we show the possible mode of action, proving a predominant anti-inflammatory response of TLR9 upon activation. There are limitations to the current study and a major caveat is the lack of evidence to show that peripheral cytokines induced by TLR9 signaling can reach and affect brain-resident microglia in vivo, which remains a major challenge in experimental design due to the species differences in TLR9 expression and cytokine profiles in human versus mouse. Future studies may consider using human iPSC-derived brain organoids with a functional blood-brain-barrier to assess the potential effects of peripheral TLR9 signaling on CNS cell types. Apart from direct effects on microglia, it is of note that the molecular machineries for cytokines (such as IFN-β) blocking IL-1β inflammatory pathway are conserved between microglia and macrophage. The current results from human iPSC-microglia also predict a similar anti-inflammatory effect of TLR9-signaling released cytokines on macrophages in the periphery. Furthermore, the effect of TLR9 induced cytokines on other cell types of the brain is still to be assessed and may disclose additional mechanisms of actions of TLR9 signaling. The current genetic findings and functional analyses together suggest a protective role of TLR9 in AD pathogenesis. We propose that loss-of-function mutations of TLR9 may disrupt a peripheral-central immune crosstalk that is required for dampening inflammation and promoting clearance of toxic protein species, and consequentially contribute to the build-up of neuroinflammation and Aβ aggregates in AD development.

Large scale genetic studies are also essential to fully understand the role of TLR9 rare variants in AD etiology. Our study is a starting point to targeted investigation, considering recent research supporting beneficial therapeutic outcomes in addition to a safety profile of TLR9 activation in in vivo models of AD [22].

### Supplementary information

Supplementary Information

Supplementary Information Excel File
