# Supplementary material for: Mutated Toll-like receptor 9 increases Alzheimer’s disease risk by compromising innate immunity protection
Source: Mol Psychiatry. 2023 Jul 11;28(12):5380–9. doi: 10.1038/s41380-023-02166-0 (PMC11041692; doi:10.1038/s41380-023-02166-0)
Supplement: Supplementary file 1 — Supplementary Information [file 41380_2023_2166_MOESM1_ESM.docx]

**Mutated** **Toll-like receptor 9 increases Alzheimer’s disease risk by compromising innate immunity protection**

Rita Cacace, PhD ^1,2^, Lujia Zhou, PhD ^3^, Elisabeth Hendrickx Van de Craen, MD ^1,2,4^, Arjan Buist, PhD ^3^, Julie Hoogmartens, PhD ^1,2^, Anne Sieben MD, PhD ^5^, Patrick Cras MD, PhD ^4,6^, Rik Vandenberghe MD, PhD ^7^, Peter P. De Deyn, MD, PhD ^2,6,8^, Daniel Oehlrich, PhD ^9^, An De Bondt, PhD ^9^, Sebastiaan Engelborghs, MD, PhD ^2,10^, Diederik Moechars, PhD ^3^ and Christine Van Broeckhoven, DSc ^1,2*^

^1^Neurodegenerative Brain Diseases, Center for Molecular Neurology, VIB, Antwerp, Belgium

^2^Department of Biomedical Sciences, University of Antwerp, Antwerp, Belgium

^3^Department of Neuroscience, Janssen Research & Development, a Division of Janssen Pharmaceutica NV, Beerse, Belgium

^4^Department of Neurology, University Hospital Antwerp, Edegem, Belgium

^5^Department of Pathology, University Hospital Antwerp, Edegem, Belgium

^6^Institute Born-Bunge, Antwerp, Belgium

^7^Department of Neurology, University Hospitals Leuven, and Department of Neurosciences, KU Leuven, Leuven, Belgium

^8^Department of Neurology and Memory Clinic, Hospital Network Antwerp, Antwerp, Belgium

^9^Discovery Sciences, Janssen Research & Development, a Division of Janssen Pharmaceutica NV, Beerse, Belgium

^10^ Department of Neurology, Universitair Ziekenhuis Brussel, and Center for Neurosciences, Vrije Universiteit Brussel, Brussels, Belgium

**Supplementary Materials and methods**

**Whole exome sequencing**

WES data of the discovery cohort, IV:3, IV:4, IV:5 and III:4, were analyzed following a dominant inheritance model. Specifically, good quality, rare, non-synonymous and splicing heterozygous variants, shared between the 4 patients were retained. Selection criteria: coverage >20x and <300x, genomic location outside simple repeats or microsatellites; clusters of variants or variants within polymorphic genes, as also described by Hartl and colleagues ^1^, were considered false positive. Minor allele frequency cut off <1% in public databases of common variants ^2^,the gnomAD database v2.1.1 ^3^ and in a database of NGS data available *in house* to exclude population specific polymorphisms ^4^.

**Chromosome 3 haplotype**

The STR markers were PCR amplified and the resulting amplicons were separated on an ABI 3730 DNA analyzer using GeneScan™ - 500 LIZ® (both ThermoFisher Scientific, MA, USA) as internal size standard. Genotype analysis was performed using the Local Genotype Viewer software v3.01 (<https://www.neuromicssupportfacility.be/>).

**Immunohistochemical examination of brain tissue of patients IV:3 and IV:5**

The brain was dissected after a formalin fixation period of approximately 8 weeks (17 days – 3.5 months), specific regions were sampled, and paraffin embedded. The following brain regions were selected, based on routine protocol. Brodmann’s area 4 (gyrus precentralis), area 6 (gyrus frontalis superior), area 8 (frontal eye fields), area 11 (gyrus rectus), area 24 (gyrus cinguli), area 22 (gyrus temporalis superior), hippocampus (level of lateral geniculate body), gyrus parahippocampalis, amygdala, area 7 (gyrus parietalis superior), area 17 (area striata), cerebellum, thalamus, neostriatum, pallidum, mesencephalon, pons, medulla oblongata.

Brain tissue from patients IV:3 and IV:5, carrying *TLR9* p.E317D mutation, are compared with two carriers of *PSEN1* mutations, p.P264L and p.I143T, two patients with sporadic AD (Iba1 staining only) and 2 control persons. Immunostaining is done with anti-CD20 (B-lymphocytes. Clone L26, Roche, Basel Switzerland), anti-CD3 (T-lymphocytes. Clone 2GV6, Roche Basel Switzerland), anti-CD68 (macrophages and microglia. Clone KP1, Agilent Technologies, CA, USA) and anti-Iba1 (microglia, clone EPR16588, ab178846, Abcam, Cambridge, UK) antibodies. The immunohistochemically stained sections of the different brain regions are semi-quantitatively rated (0: no abnormalities; +, ++, +++) by 2 independent blinded neuropathologists (JJM and AS) for CD68, CD20 and CD3 and by 3 blinded neuropathologists for Iba1 (LM, AM, SA). The evaluation of the microglia was performed based on the recent publication by Schwabenland and colleagues ^5^ in which the assessment is based on a multistep process: 1) Identification of myeloid cells in the CNS: as perivascular macrophages are also Iba1 immunoreactive, perivascular Iba1 positive cells were excluded; 2) Cell density: an increase of the number of microglial cells. Only the presence of immunoreactive soma with nucleus were included. Immunoreactive processes without associated soma were not considered; 3) Cell shape: the form of the soma and processes was evaluated and 4) Distribution pattern: focal accumulation and microglial territories.

**Transcript analysis by nonsense mediated decay assay**

Non-sense mediated mRNA decay (NMD) was inhibited in lymphoblastoid cell line (LCL) of family members, both C-C chemokine receptor type 3 (*CCR3*) frameshift variants carriers (*CCR3* p.F249Hfs*23, rs561062190, n=3) and one non-carrier. Specifically, 5 x 10^6^ LCL were treated with either 150µg/ml of cycloheximide or vehicle (DMSO) for 4 hours at 37°C. After the incubation, RNA was isolated using the RiboPure™ kit followed by DNase treatment with TURBO DNase (both Ambion, ThermoFisher Scientific, MA, USA). First-strand cDNA was synthetized utilizing the SuperScript® III First-Strand Synthesis System (ThermoFisher Scientific, MA, USA) with random hexamer primers. PCR amplification of the 3’ region of *CCR3* was achieved with specific primers (available upon request) followed by Sanger sequencing (BigDye Terminator Cycle Sequencing kit v3.1; analysis on an ABI 3730 DNA Analyzer, both ThermoFisher Scientific, MA, USA). Electropherograms analysis was performed in SeqMan (DNASTAR, Madison, WI, USA).

**RNA and protein expression of TLR9 in LCL**

RNA was extracted as outlined above. RT-qPCR reactions were performed using the Fast SYBR® Green chemistry (Thermo Fisher Scientific, MA, USA) and run on the **xxx**

Real-Time PCR System (Thermo Fisher Scientific, MA, USA). Relative quantification of TLR9 mRNA levels was achieved with glyceraldehyde 3-phosphate dehydrogenase (GAPDH), tyrosine 3-monooxygenase/tryptophan 5-monooxygenase activation protein, zeta polypeptide (YWHAZ), hypoxanthine phosphoribosyltransferase 1 (HPRT1), TATA box binding protein (TBP) genes and beta 2 microglobulin (B2M) as reference genes. Protein lysates were made, as previously described ^6^, in modified radioimmunoprecipitation (RIPA) buffer (150 mM NaCl, 0.5% sodium deoxycholate, 1% NP-40, 50 mM Tris-HCl; pH 8.0) supplemented with 1% sodium dodecyl sulfate (SDS) ^7^ and phosphatases and proteases inhibitors (PhosSTOP™ (Sigma-Aldrich, MO, USA) and cOmplete™ Protease Inhibitor Cocktail (Roche, Basel, Switzerland)). Protein preparations were separated on 4-12% NuPAGE® Bis-Tris gel (ThermoFisher Scientific, MA, USA) and electroblotted onto a polyvinylidene difluoride membrane (PVDF, Hybond P; Amersham Biosciences, GE Healthcare Life Sciences, Buckinghamshire, UK). Membranes were probed with primary antibodies to detect TLR9 (1:5000 Abcam, ab187148, Cambridge, UK) and GAPDH (1:20000 GeneTex, GTX100118, CA, USA). Immunodetection was performed with specific secondary antibodies conjugated with horseradish peroxidase (HRP) and the ECL-plus chemiluminescent detection system (GE Healthcare Life Sciences). Western blot results were visualized using Amersham Imager 680 (Cytiva, MA, USA).

**TLR9 receptor activation by NF-κB luciferase-based assay for variants modelling**

To generate the plasmid, human *TLR9* gene was cloned behind the CMV promoter into the multiple cloning site of the pcDNA5/FRT plasmid (ThermoFisher Scientific, MA, USA) and subsequently the NlucP (NanoLuc-PEST) reporter gene behind a minimal promoter with 5 NF-κB response elements was restricted with SpeI and BamHI from the pNL3.2.NF-κB-RE[NlucP/NF-κB-RE/Hygro] vector (Promega, WI, USA) and cloned into the same TLR9-pcDNA5/FRT plasmid. *TLR9* variants were generated by site-directed mutagenesis (ThermoFisher Scientific, MA, USA). For the generation of isogenic stable lines the TLR9/NlucP-pcDNA5/FRT plasmid with TLR9 wild type or TLR9-E317D were co-transfected with pOG44 (ThermoFisher Scientific, MA, USA) expressing the Flp-recombinase using the Lipofectamine™ 2000 reagent (ThermoFisher Scientific MA, USA) according to the recommendations of the supplier into the Flp-In™-293 cell Line (ThermoFisher Scientific, MA, USA). These cells contain a single stably integrated FRT site at a transcriptionally active genomic locus. Monoclonal cell lines were isolated by limited dilution cloning after selection with 200 µg/ml Hygromycin B. Individual clones have been verified using TLA sequencing of monoclonal lines by Cergentis (Utrecht, The Netherlands). To perform the TLR9 reporter assay, HEK293 cells, transfected with the plasmid expressing both the TLR9 variant under a CMV promotor and the destabilized form of NanoLuc luciferase reporter driven by an NF-κB response element, 5000 cells/well were seeded in 384-well white bottom plates and stimulated with different concentrations of CpG-ODN or with 0.1 ng/µl TNF-α (Sigma-Aldrich, MO, USA). The remaining cells were kept for TLR9 protein expression analysis. Fumarizine (Promega, WI, USA) was added 6 hours after stimulation and the luminescence was measured in a PerkinElmer EnVision plate reader. Luminescence was calculated relative to the TNF-α response in the same cell line and analyzed with the GraphPad Prism software.

**RNA extraction and RT-qPCR**

RNA extraction of human iPSC-derived microglia was performed using the RNeasy plus mini kit (Qiagen, Germany) following manufacture’s protocol and RNA concentration was determined by Nanodrop measurement. For RT-qPCR analysis, RNA extraction was subjected to cDNA synthesis using SuperScript® III (Life Technologies, CA, USA) followed by RT-qPCR using IDT TaqMan assays to detect TLR9 (Hs.PT.58.40576968), TYROBP (Hs.PT.58.38520183.g), TREM2 (Hs.PT.58.40294042), GPR34 (Hs.PT.58.3531094). IDT TaqMan assays for POLR2A (Hs.PT.39a.19639531) and TBP (Hs.PT.58v.39858774) were used as housekeeping references. RT-qPCR data were analyzed using Qbase+ software (Biogazelle, Belgium).

**PBMCs preparation and treatment**

Human peripheral blood mononuclear cells (PBMCs) were prepared using ACCUSPINTM System-Histopaque®-1077 (Sigma-Aldrich, MO, USA) following manufacture’s protocol. Briefly, blood samples were collected from healthy donors and diluted 2-fold in PBS. Diluted blood samples were added into ACCUSPIN tube and centrifuged at 800 x g for 15 minutes with brake off. After centrifugation, the opaque interface was transferred to a clean 50 mL conical centrifuge tube. The cells were washed twice with PBS and centrifuged at 250 x g for 10 minutes each time. Cell pellets were re-suspended in RPMI medium (Gibco, ThermoFisher Scientific, MA, USA) supplemented with 10% heat inactivated FBS (Biowest, France) and 100 U/ml Penicillin-Streptomycin. For treatment, PBMCs are plated on either 96-well cell culture microplates with 500,000 cells per well, or 12-well cell culture plates with 5 million cells per well. After plating, cells are treated with 1 μM TLR9 agonist ODN2216 (InvivoGen, CA, USA), 2.5 μM TLR8 agonist (Janssen, JNJB39224507) or 8.33 μM TLR7 agonist (Janssen, JNJB43025409). Conditioned media were collected 24 hours after compound treatment and subjected to Luminex multiplex assays or used for other functional assays. The working concentrations of different agonists were selected based on their individual potencies. A selective TLR9 antagonist (Janssen, JNJB35419342) was used at 500 nM to confirm the specificity of TLR9 agonist in the first pilot experiments.

**Microglia Differentiation**

Human induced pluripotent stem cells (iPSCs) were differentiated to microglia following previously described protocol ^8^. Briefly, human iPSCs were split using TrypLETM Select (Life Technologies, CA, USA) and seeded into an AggreWellTM800 24-well plate (Stemcell Technologies, Vancouver, Canada) with approximately 2.5 x 10^6^ cells per well for embryoid bodies (EBs) formation in daily-refreshed mTeSR1 medium supplemented with 50 ng/mL BMP4 (Invitrogen, CA, USA), 50 ng/mL VEGF (Peprotech, NJ, USA), and 20 ng/mL SCF (Miltenyi Biotec, Germany). After three-to-four days, EBs were filtered through 37-μm reversible cell strainer (Stem Cell Technologies, Vancouver, Canada) and placed into 6-well plates (15 EBs/well) for differentiation into macrophage precursors in X-VIVO15 (Lonza, Basel, Switzerland), supplemented with 100 ng/mL M-CSF (Invitrogen, CA, USA), 25 ng/mL IL-3 (ThermoFisher Scientific, MA, USA), 2 mM Glutamax (ThermoFisher Scientific, MA, USA), 100 U/ml Penicillin-Streptomycin (Gibco, ThermoFisher Scientific, MA, USA), and 50 μM β-mercaptoethanol (ThermoFisher Scientific, MA, USA), with half medium change weekly. After approximately 6 weeks, macrophage precursors emerged into the supernatant and were collected weekly with half-medium replenishment. For microglia differentiation, precursor cells were harvested via filtering through 37-μm reversible cell strainer and plated into 96-well cell culture microplates (Greiner Bio-one, Austria) with 18,000 cells per well in Advanced DMEM/F12 supplemented with 100 ng/mL IL-34, 10 ng/mL GM-CSF, 1 mM Glutamax, 100 U/ml Penicillin-Streptomycin, and 50 μM β-mercaptoethanol, with half medium change twice per week. Cells were differentiated for 14 to 18 days before assaying.

**Phagocytosis assays**

Aβ_42_ oligomers (Crossbeta Biosciences, Utrecht, The Nederlands) were labeled with pHrodo™ iFL Red STP Ester (ThermoFisher Scientific, MA, USA) following manufacturer's protocol, and were dialyzed using 10 kDa Slide-A-Lyzer™ Dialysis Cassettes (ThermoFisher Scientific, MA, USA) in ice-cold PBS to remove free dyes. To measure phagocytosis, human iPSC-derived microglia cells cultured in 96-well microplates were incubated with 1 μg/mL pHrodo-Aβ_42_ diluted in culture medium for 4 hours, and further incubated with HSC CellMask™ Deep Red stain (ThermoFisher Scientific, MA, USA) at 1:5000 dilution for 5 minutes before live-cell scanning using Perkin Elmer Opera Phenix. Images were analyzed using Harmony high-content imaging and analysis software (PerkinElmer, version 4.1). Phagocytosis of pHrodo-Aβ_42_ were calculated by dividing total fluorescence intensities of pHrodo by the number of cells quantified from CellMask stain.

To assess the effects of individual cytokine or mixture of cytokines induced by TLR9-signaling, microglia cells were pre-treated with 50 ng/ml recombinant IFNα-2a (ProSpec, Israel), IFN-β (PBL Assay Science, NJ, USA), IFN-λ1 (R&D systems, MN, USA), IFN-γ (R&D systems, MN, USA), IL-1RA (ProSpec, Israel), IL-10 (Gibco, ThermoFisher Scientific, MA, USA), SCF (Miltenyi Biotec, Germany), or 10-fold diluted PBMCs conditioned medium for approximately 14 hours before phagocytosis assay.

**Phagocytosis assays with pHrodo-Zymosan**

Human iPSC-derived microglia cells cultured in 96-well microplates were pre-treated with 50 ng/ml recombinant IFN-β (PBL Assay Science, NJ, USA) or IFN-γ (R&D systems, MN, USA) for approximately 14 hours preceding phagocytosis assay. To perform the phagocytosis assay, microglia cells were incubated with 100 μg/mL pHrodo-Zymosan (ThermoFisher P35364) diluted in culture medium for 4 hours, and further incubated with HSC CellMask™ Deep Red stain (ThermoFisher Scientific, MA, USA) at 1:5000 dilution for 5 minutes before live-cell scanning using Perkin Elmer Opera Phenix. Images were analyzed using Harmony high-content imaging and analysis software (PerkinElmer, version 4.1). Phagocytosis of pHrodo-Zymosan were calculated by dividing total fluorescence intensities of pHrodo by the number of cells quantified from CellMask stain.

**Induction of IL-1β release from microglia**

Induction of IL-1β release *in vitro* followed previously described protocol ^9^. Briefly, human iPSC-derived microglia were primed with 10 ng/mL ultrapure LPS (Invivogen, CA,, USA) for 4 hours preceding inflammasome stimulation with 10 μM nigericin (Merck Millipore, MA, USA) for 2 hours. The supernatants were collected for cytokine measurement by MSD multiplex assay (Meso Scale Discovery, MD, USA). Cells were lysed in RIPA (Sigma-Aldrich, MO, USA) supplemented with cOmplete™ ULTRA Protease Inhibitor Cocktail (Sigma-Aldrich, MO, USA), and subjected to CellTiter-Glo® Luminescent Cell Viability Assay (Promega, WI, USA) following manufacturer's protocol.

To assess the effects of IFNβ or mixture of cytokines induced by TLR9-signaling on IL-1β release, microglia cells were pre-treated with dilutions of IFNβ, or 10-fold diluted PBMCs conditioned media for approximately 14 hours preceding LPS priming.

**Microarray analysis**

RNA extraction was prepared with the RNeasy plus mini kit (Qiagen, Germany) for microarray analysis. Amplification and labelling of total RNA were performed using GeneChip® 3’ IVT Express Kit following manufacture’s protocol (Affymetrix 2004, CA, USA). Biotin-labeled target samples were hybridized to the GeneChip® Clariom_S_Human_HT containing probes for over 18k genes. Target hybridization was processed on the GeneTitan® Instrument according to manufacturer’s instructions provided for Expression Array Plates (P/N 702933). Images were analysed using the GeneChip® Command Console Software (AGCC) (Affymetrix, CA, USA). Microarray data were processed using the statistical computing R-program (R version 3.4.2) and Bioconductor tools ^10^. The gene expression values were normalized using Robust Multi-array verage (RMA) ^11^. Individual probes were grouped into gene-specific probe sets based on Entrez Gene using the metadata package clariomshumanhthsentrezg (version 22.0.0) ^12^.

**Supplementary Results**

| **ID** | **AAO** | **AAD** | **DD** | **Diagnosis** | **APOE** | **Signs & symptoms** | **Imaging** |
| --- | --- | --- | --- | --- | --- | --- | --- |
| II:5 | NA | 83 | NA | Possible AD | NA | Amnestic presentation with further decline to dementia | NA |
| III:4 | NA | 90 | NA | Possible AD | ε3ε3 | Amnestic presentation, extrapyramidal syndrome, visual hallucinations | NA |
| III:9 | 64 | 76 | 12 | Possible AD | NA | Amnestic presentation with behavioral problems | CT: corticosubcortical atrophy (L>R) |
| IV:3 | 52 | 63 | 11 | Definite AD (A3B2-3C3) | ε3ε3 | Amnestic presentation with dyscalculia.  Important visuo-spatial and visuo-constructive dysfunction, mildly diminished abstract reasoning and disorientation. Frontal release signs are present.  Further cognitive decline: concentration difficulties, executive dysfunction, anomia, dyspraxia. Mild parkinsonism in left hemicorpus at end-stage. BPSD (motoric restlessness). | MRI: corticosubcortical atrophy with extensive periventricular leuko-encephalopathy.  SPECT: PT (R>L) hypoperfusion as well as T + O (L+R), F (R), O (L+R) |
| IV:4 | 55 | 60 | 5 | Probable AD | ε4ε4 | Amnestic presentation.  Severe episodic memory dysfunction with moderate dysexecutive function, concentration dysfunction and apraxia. Desorientation, anomia, aphasia. | MRI: cortical atrophy  SPECT: relative hypoperfusion P (R>L) with mild hypoperfusion T+F+O (R>L) and CB (L) hypoperfusion |
| IV:5 | 60 | 69 | 9 | Definite AD (A3B3C3) | ε4ε4 | Amnestic presentation  Anomia, change in personality (anxiety), visuo-constructive dysfunction, episodic recent memory dysfunction. | CT + MRI: normal  SPECT: mild aspecific cortical hypoperfusion |
| IV:7 | 58 | 69 | 11 | Probable AD | ε3ε4 | Amnestic presentation with orientation abnormalities. In later stages severe behavioral changes were seen. | CT: corticosubcortical atrophy |
| **Average**  **StDev** | **57.8**  **4.6** | **72.9 10.8** | **9.6 2.8** |  |  |  |  |
| IV:2 | 68 | NA | NA | Subjective cognitive decline | ε3ε4 | Short-term memory problems and wordfinding disorders. MMSE 30/30 | NA |

**SI Table 1: Clinical characteristics of AD patients.** Abbreviations: AAO = age at onset, AAD = age at death, DD = disease duration, AD = Alzheimer’s disease, VAD = Vascular dementia, MRI = magnetic resonance imaging, SPECT = Single-photon emission computed tomography, CT = computed tomography, PT = parietotemporal, T = temporal, O = occipital, F = frontal, CB = cerebellar, L= left, R= right, NA= not accessible. Patients II:5 and III:9 no DNA available.

| **ID** | ***Current age*/AAD** | **APOE** |
| --- | --- | --- |
| III:1 | 85 | ε3ε3 |
| III:2 | 84 | ε2ε3 |
| III:3 | 85 | ε3ε4 |
| III:5* | 90 | NA |
| III:6* | 79 | ε3ε4 |
| III:7 | 73 | ε3ε4 |
| III:8^#^ | 73 | ε3ε3 |
| IV:1 | 63 | ε3ε3 |
| IV:6 | 62 | ε3ε4 |
| **Average**  **StDev** | **75.5**  **9.4** |  |

**SI Table 2: Clinical characteristics of at risk or healthy family members.**

AAD = age at death.

*Note: III:5 did not undergo in depth neurological examination. A cognitive decline five years prior death was mentioned by first degree relative, no DNA available. III:6, suffered from a subarachnoidal bleeding (SAB) as well as 2 ischemic cerebrovascular accidents (iCVA). MRI, showed corticosubcortical atrophy. Further, there was an occlusion of the left internal carotid artery (ICA) and stenosis of the right ICA, which is probably the cause of the iCVA’s. #III:8: no clinical information could be retrieved, disease status unknown.

| **Gene** | **gDNA (g.)** | **Transcript (NM_)** | **cDNA (c.)** | **Protein (p.)** | **dbSNP** | **HEX** | **gnomAD MAF%** |
| --- | --- | --- | --- | --- | --- | --- | --- |
| *CCR3* | g.46307387_46307389 | NM_178329.3 | c.738delGT | p.F249Hfs*23 | rs561062190 | - | 0.0000088 |
| *ZNF589* | g.48310245 | NM_016089.3 | c.1064C>T | p.T355M | rs376706270 | - | 0.000015 |
| *TLR9* | g.52257381 | NM_017442.3 | c.951G>C | p.E317D | - | - | - |

**SI Table 3: Summary of the 3 variants co-segregating in the family.** Genomic position (g.) is based on reference genome GRCh37/hg19. None of the co-segregating variants was found in the HEX database (<https://www.alzforum.org/exomes/hex>), exome sequencing database of 478 neuropathological healthy controls with an age at inclusion over 60 years. The variants in *CCR3* and *ZNF589* are ultra-rare while the TLR9 p.E317D is novel. MAF = minor allele frequency in non-Finnish European (v2.1.1 and v3 lastly accessed in June 2020).

| **STR marker** | **cM** | **Disease alleles (bp)** | **Disease allele Freq (%)** | **N screened controls** |
| --- | --- | --- | --- | --- |
| D3S3727 | 56.07 |  |  |  |
| D3S2432 | 57.92 | 336 | 28.3 | 274 |
| D3S1619 | 60.98 | 248 | 19.6 | 337 |
| D3S2407 | 67.94 | 239 | 25.3 | 336 |
| D3S3559 | 67.94 | 249 | 23.1 | 321 |
| D3S3647 | 68.47 | 234 | 37.5 | 336 |
| D3S3582 | 69.19 | 164 | 43 | 342 |
| ***CCR3*** | **GT** | **delGT** |  |  |
| D3S1767 | 69.9 | 387 | 42.7 | 327 |
| D3S2420 | 70.61 | 107 | 7.8 | 340 |
| ***ZNF589*** | **C** | **T** |  |  |
| D3S3629 | 70.61 | 249 | 14.2 | 334 |
| D3S3026 | 70.61 | 210 | 17.3 | 347 |
| ***TLR9*** | **C** | **G** |  |  |
| D3S1289 | 71.41 |  |  |  |
| D3S2408 | 74.35 |  |  |  |
| D3S1300 | 80.32 |  |  |  |

**SI Table 4: Chromosome 3 short tandem repeats (STR) alleles genotypes and frequency.** STR markers are as in the Marshfield map. The disease alleles identified by family analysis are reported in base pairs (bp). The frequency of the disease alleles was determined genotyping a cohort of controls. The number of control individuals genotyped is also reported (range 264 – 347 individuals). The double line delineates disease haplotype based on the chromosomal recombinations identified in patient IV:5, between markers D7S3727 and D3S2432, and patient IV:3, between markers D3S3026 and D3S1289. The disease haplotype size, delimited by D3S3727 and D3S1289, is 15.34 cM, equivalent to 23.8 Mb on chr3p24.1-p14.3.


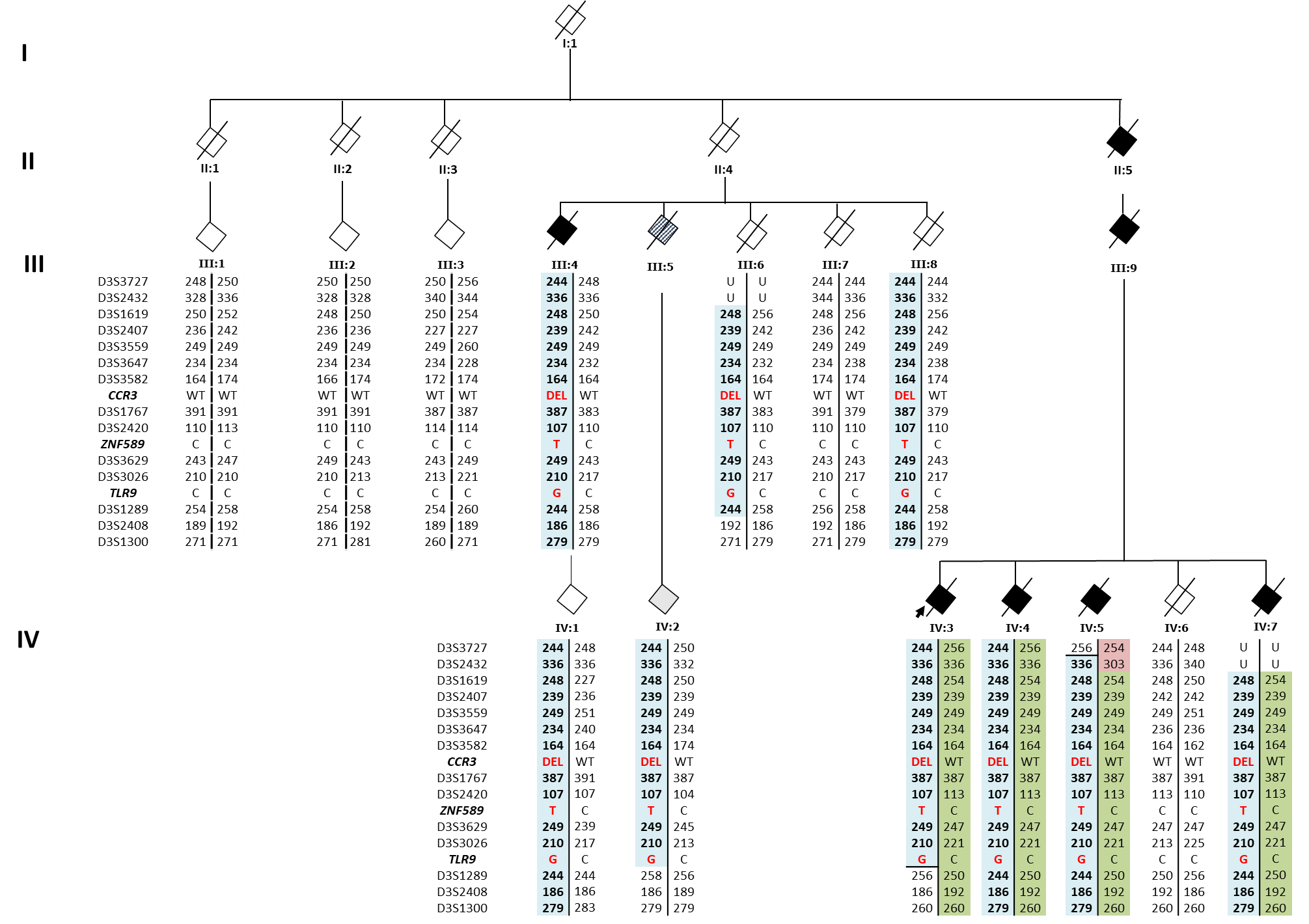


**SI Figure 1: Complete haplotype analysis in the family.** Shared haplotype co-segregating on chr3p24.1-p14.3. Two recombination events are reported: in patient IV:5 (markers D7S3727 and D3S2432), and in patient IV:3 (markers D3S3026 and D3S1289).

| **Marker** | **IV:3** | |  | **DR1578** | |
| --- | --- | --- | --- | --- | --- |
| D3S3727 | 244 | 256 |  | 250 | 250 |
| D3S2432 | 336 | 336 |  | 332 | 340 |
| D3S1619 | 248 | 254 |  | 248 | 256 |
| D3S2407 | 239 | 239 |  | 239 | 230 |
| D3S3559 | 249 | 249 |  | 249 | 255 |
| D3S3647 | 234 | 234 |  | 234 | 232 |
| D3S3582 | 164 | 164 |  | 164 | 174 |
| ***CCR3*** | **delGT** | WT |  | **delGT** | WT |
| D3S1767 | 387 | 387 |  | 387 | 383 |
| D3S2420 | 107 | 113 |  | 107 | 116 |
| ***ZNF589*** | **T** | C |  | **T** | C |
| D3S3629 | 249 | 247 |  | 245 | 251 |
| D3S3026 | 210 | 221 |  | 213 | 221 |
| ***TLR9*** | G | C |  | C | C |
| D3S1289 | 256 | 250 |  | 258 | 256 |
| D3S2408 | 186 | 192 |  | 186 | 198 |
| D3S1300 | 260 | 260 |  | 260 | 281 |

**SI Table 5: Allelic-sharing analysis between IV:3 and DR1578.** DR1578 is a control person carrying both p.F249Hfs*23 and the *ZNF589* p.T355M variants. Allelic sharing analysis with IV:3, shows a recombination event between the *ZNF589* gene and D3S3629 marker with the exclusion of *TLR9* gene. DR1578 presented an additional recombination between D3S2432 and D3S1619.


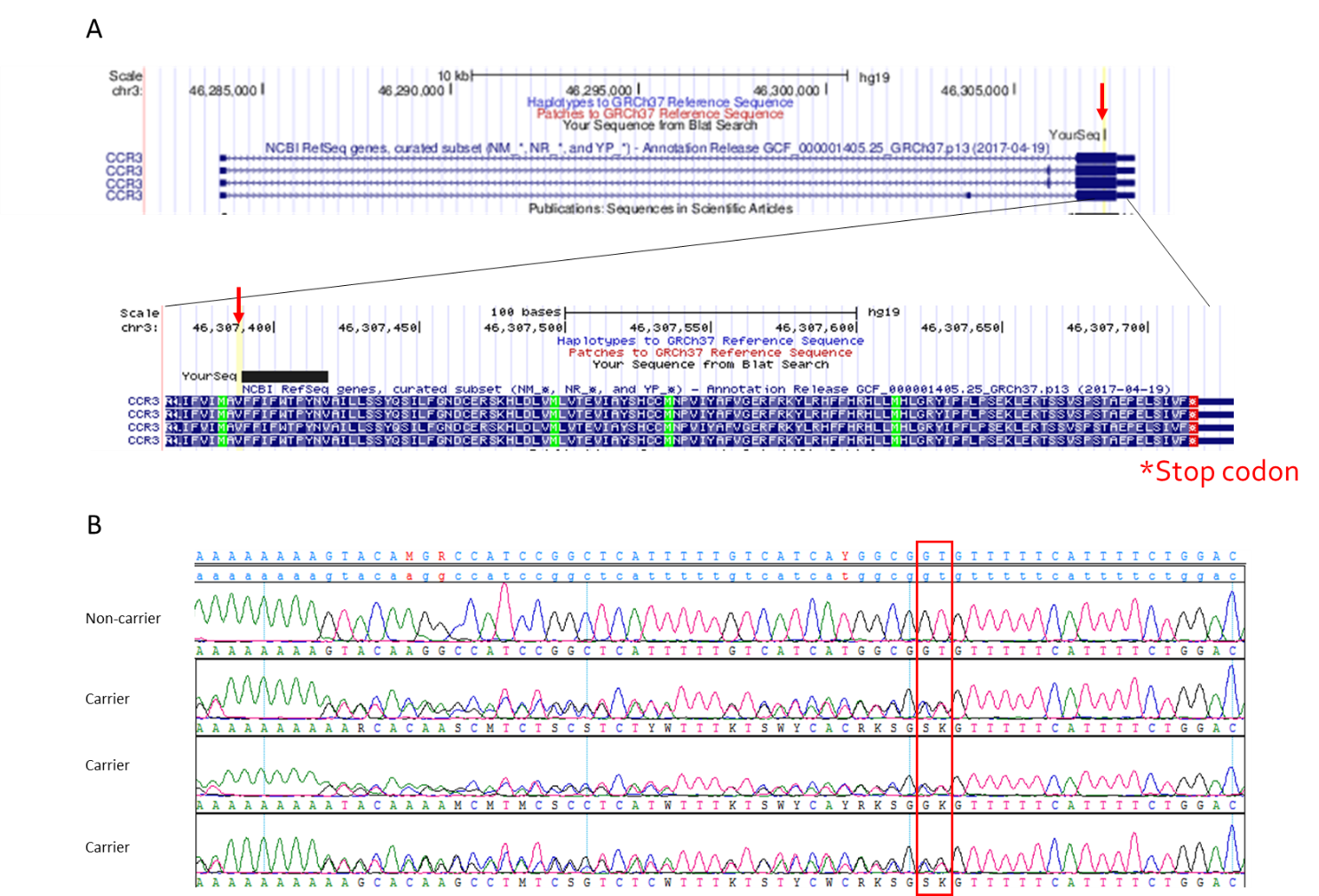


**SI Figure 2: *CCR3* c.738delGT mutated transcript escapes non-sense mediated decay (NMD).** A) Genomic location of *CCR3* delGT frameshift variant as from UCSC genome browser (GRCh37/hg19). The red arrow marks the position of the dinucleotide deletion in the last exon of *CCR3* transcript. B) NMD results, electropherogram traces of a non-carrier (first trace) and 3 carriers from the family from the reverse sequence. The red rectangle marks the c.738delGT. The presence of the mutated transcript, in overlap with the wild type allele in the last 3 traces, shows NMD escape, which is not unexpected due to the location of the mutation in the last exon of the gene.


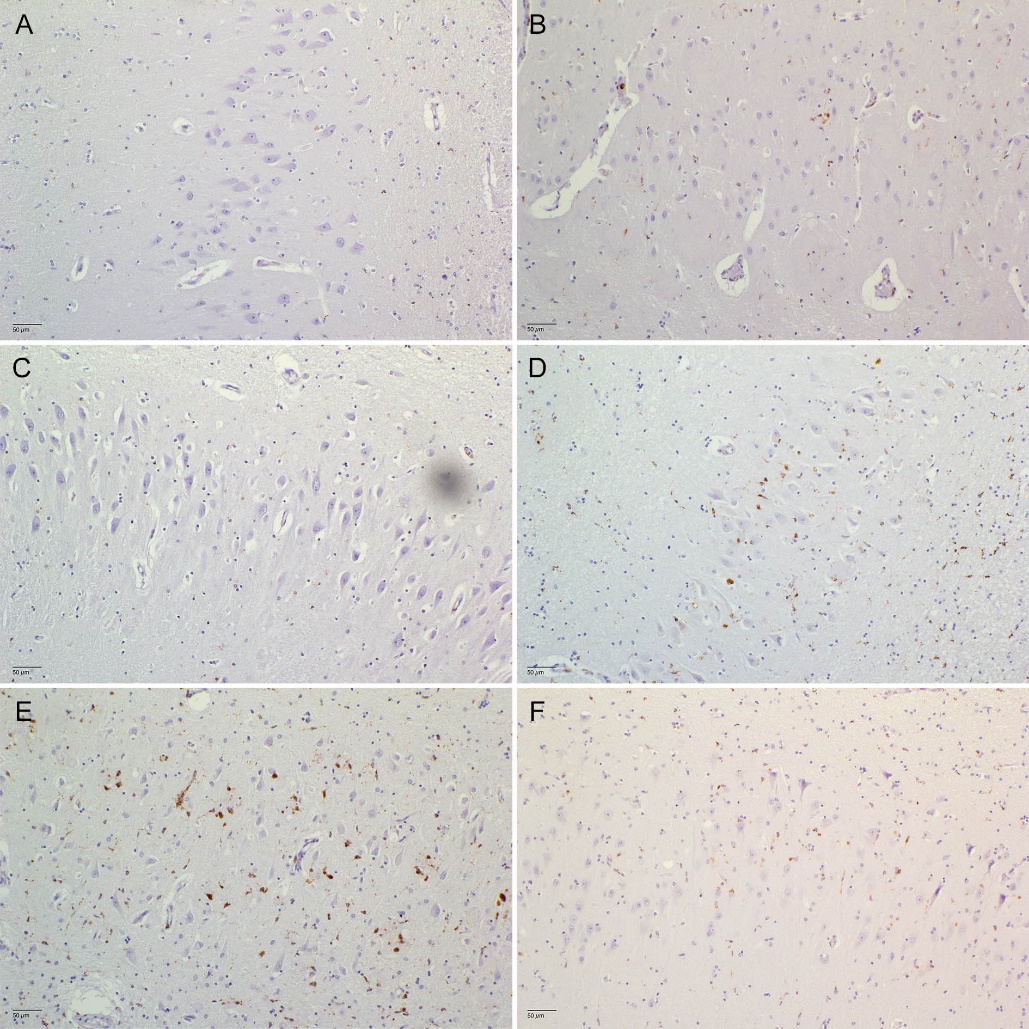


**SI Figure 3: CD68 immunostaining of hippocampal dentate gyrus.** A) and C) controls, B) *PSEN1* p.I143T; D) IV:5 (TLR9 p.E317D); E) *PSEN1* p.P264L and F) IV:3. Note the sparse CD68 immunoreactivity in the white matter, and absent immunoreactivity in cortex of A & C. In the *PSEN1* and *TLR9* cases, there is an increased immunoreactivity in white matter and cortex.


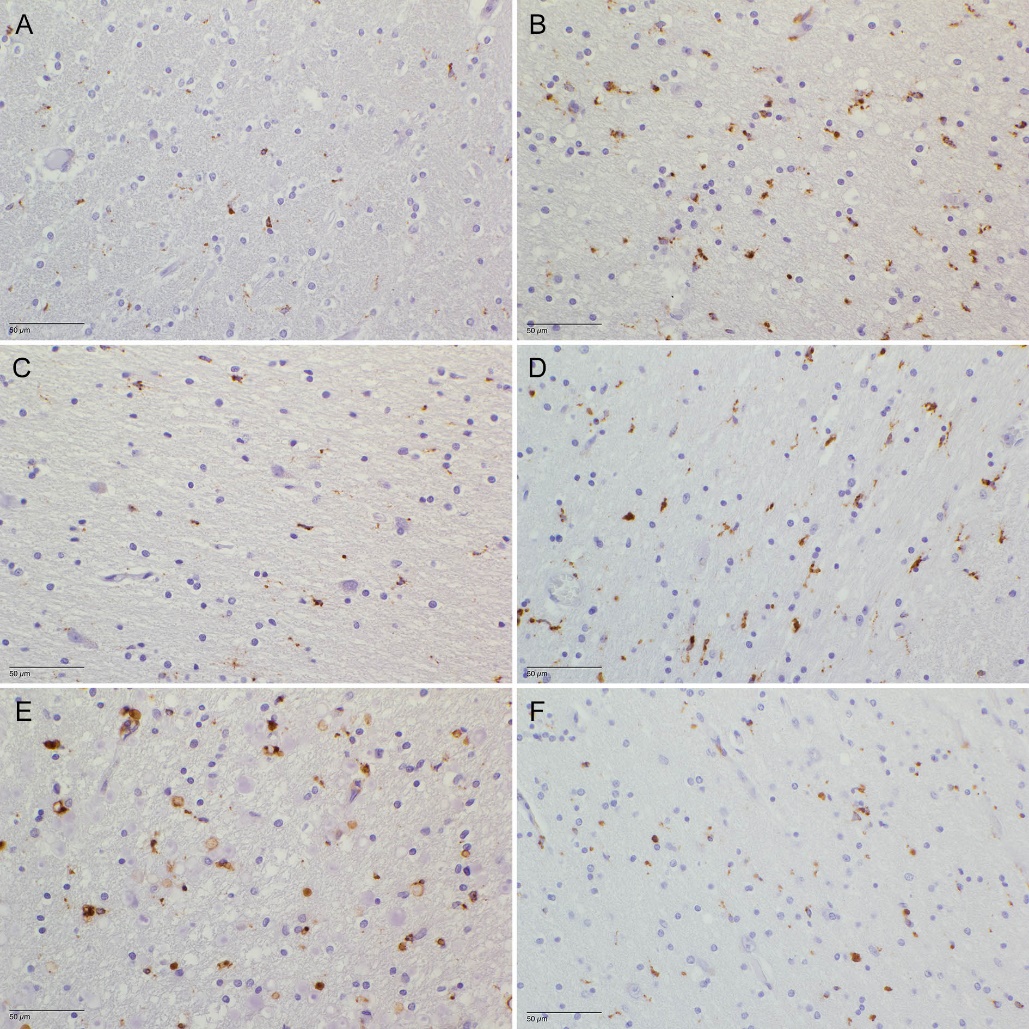


**SI Figure 4: CD68 immunostaining of hippocampus. Greater magnification.** A) and C) controls, B) *PSEN1* p.I143T; D) IV:5 (TLR9 p.E317D); E) *PSEN1* p.P264L and F) IV:3 (TLR9 p.E317D). No immunoreactivity in the controls (A and C) whereas in the *PSEN1* carriers (B and E) and in the *TLR9* carriers (D and F) a high number of macrophages and microglia was found.

| **CD68** | **IV:3** | **IV:5** | ***PSEN1* p.I143T** | ***PSEN1* p.P264L** |
| --- | --- | --- | --- | --- |
| Area 10 (prefrontal) | + | ++ | + | ++ |
| Hippocampus | + | ++ | + | ++ |
| Amygdala | NA | ++ | + | ++ |
| Mesencephalon | + | ++ | + | ++ |
| Cerebellum | + | ++ | + | ++ |
| Area Striata | + | ++ | + | ++ |
| Area 22 (temporal neocortex) | + | ++ | + | ++ |
| Lesion load | 4 | 1 | 3 | 2 |

**SI Table 6: Semi-quantitative rating of CD68 positivity in TLR9 and PSEN1 carriers.** The most severe CD68 immunoreactivity was seen in IV:5, followed by *PSEN1* p.P264L than *PSEN1* p.I143T and IV:3. Lesion load is scored in a scale from 1 = most severe to 4 least severe.


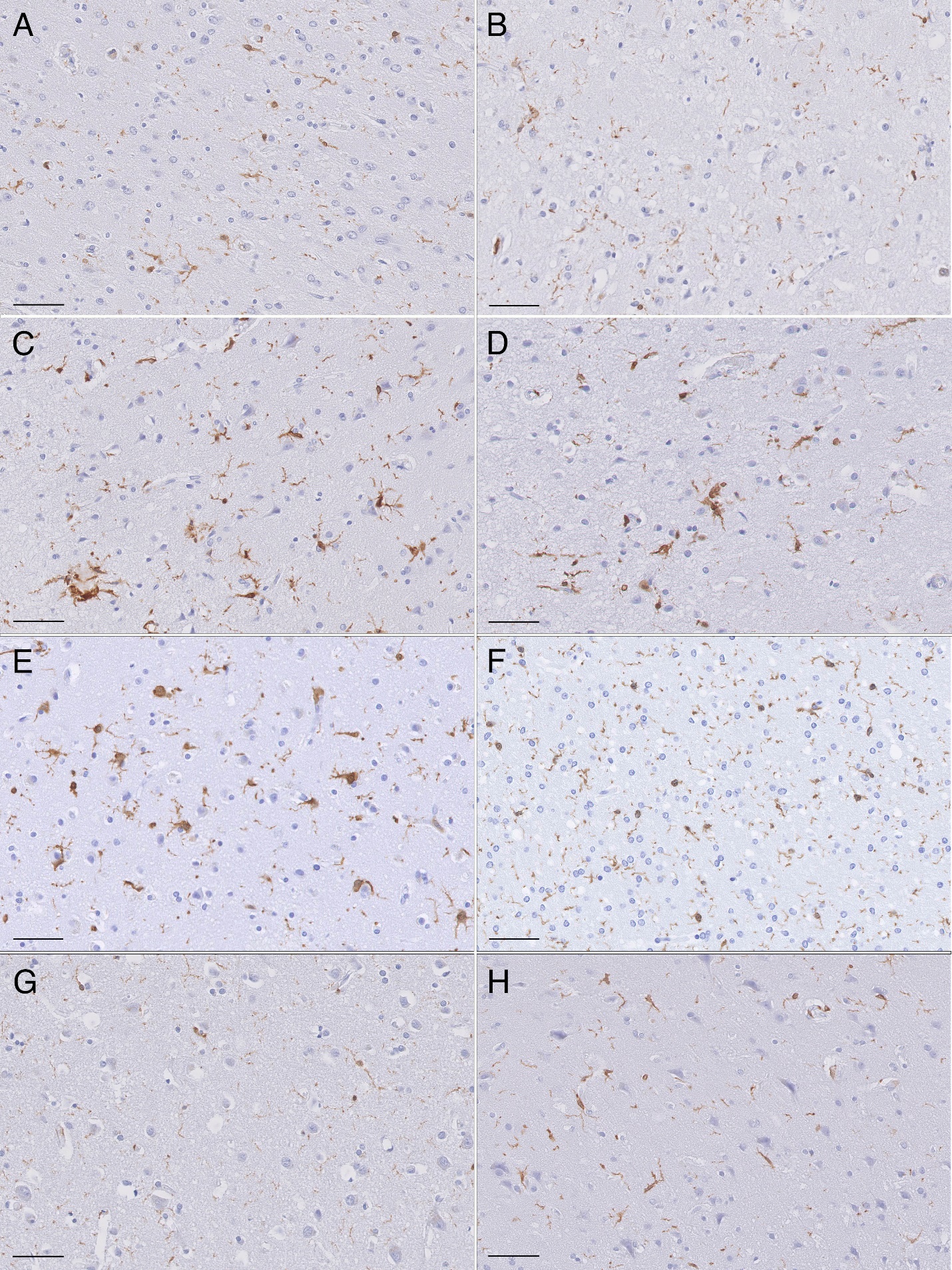


**SI Figure 5**: **Iba1 staining of frontal cortex.** Scale bar 50 μm. A) IV:5 and B) IV:3; both patients carry the TRL9 p.E317D variant, C) and D) are AD patients with *PSEN*1 p.P264L and p.I143T respectively, E) and F) are two sporadic AD patients and G) and H) are neurologically healthy controls.

**A**

*
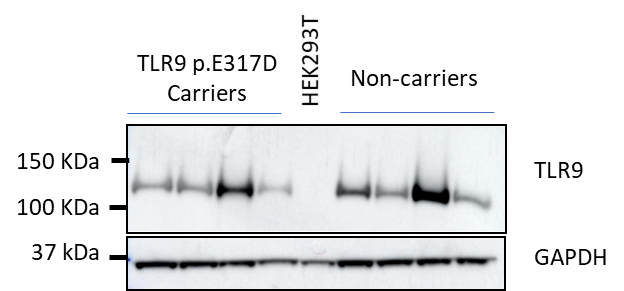
*

**B**

**SI Figure 6:**  **TLR9 expression studies** in LCL of p.E317D carriers (n=4) and non-carriers (n=4) from the family. TLR9 RNA (A) and protein (B) expression are not affected by the p.E317D mutation in LCL.

**SI Figure 7: E317D co-segregating variant decreases TLR9 signaling.** Isogenic HEK293 cells expressing TLR9 wild type or TLR9-E317D. TLR9 stimulation occurred with different concentrations of the CpG ODN2006 or with 0.1 ng/µl TNF-α, which was used as control for expression levels. Luminescence is expressed relative to the TNF-α response in the same cell line with the TLR9 wild type response set at 100%. The average of 3 independent experiments is shown.


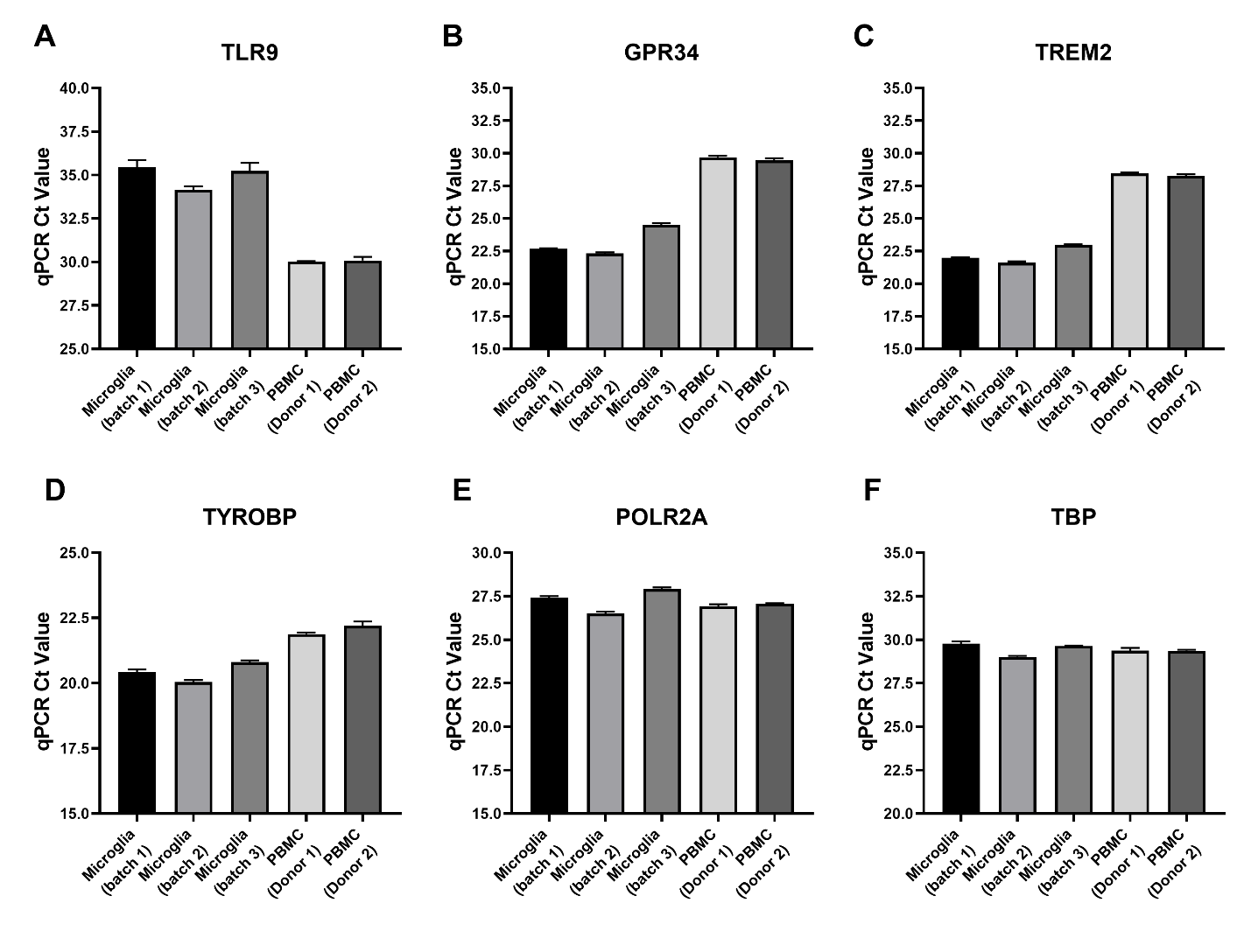


**SI Figure 8:** **RT-qPCR analysis of TLR9 expression in human iPSC-derived microglia.** RNA samples from human iPSC-derived microglial cells (batch 1, batch 2 and batch 3 from three independent differentiation) were analyzed by RT-qPCR, with RNA samples from human PBMCs (donor 1 and donor 2) included as controls. The Ct values are plotted as mean ± SEM, n=3 technical replicates. Note TLR9 mRNA is barely expressed in human iPSC-derived microglia cells (Ct values are above 35) but is expressed in human PBMCs (Ct values ~30). In contrast, microglial markers including GPR34, TREM2 and TYROBP are highly expressed in human iPSC-derived microglial cells (Ct values ~20-25). Housekeeping genes including POLR2A and TBP are equally expressed in human iPSC-derived microglia cells versus human PBMCs.

**
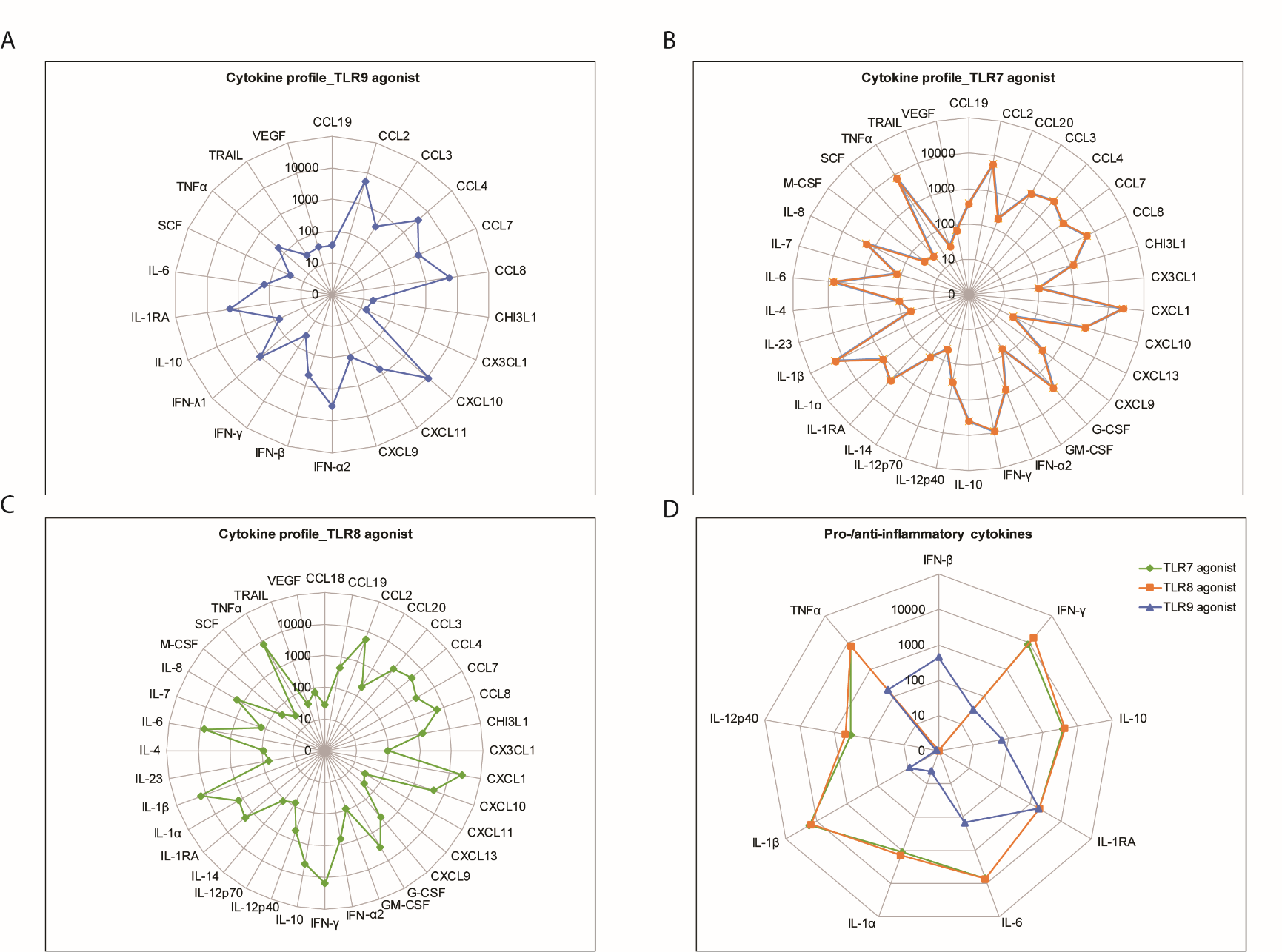
**

**SI Figure 9: Cytokine profiles of human PBMCs in response to TLR9, TLR7 or TLR8 agonist.** PBMCs prepared from one healthy donor were treated with (A) 1 μM TLR9 agonist ODN2216, (B) 2.5 μM TLR8 agonist JNJB39224507, or (C) 8.33 μM TLR7 agonist JNJB43025109 for 24 hours, conditioned media were collected and subjected to Luminex multiplex assays. The concentrations of individual cytokines are background subtracted and plotted on radar chart, axis unit pg/mL. (C) Comparison of pro- and anti- inflammatory cytokines released from PBMCs in response to different TLR agonists is plotted in a radar chart.


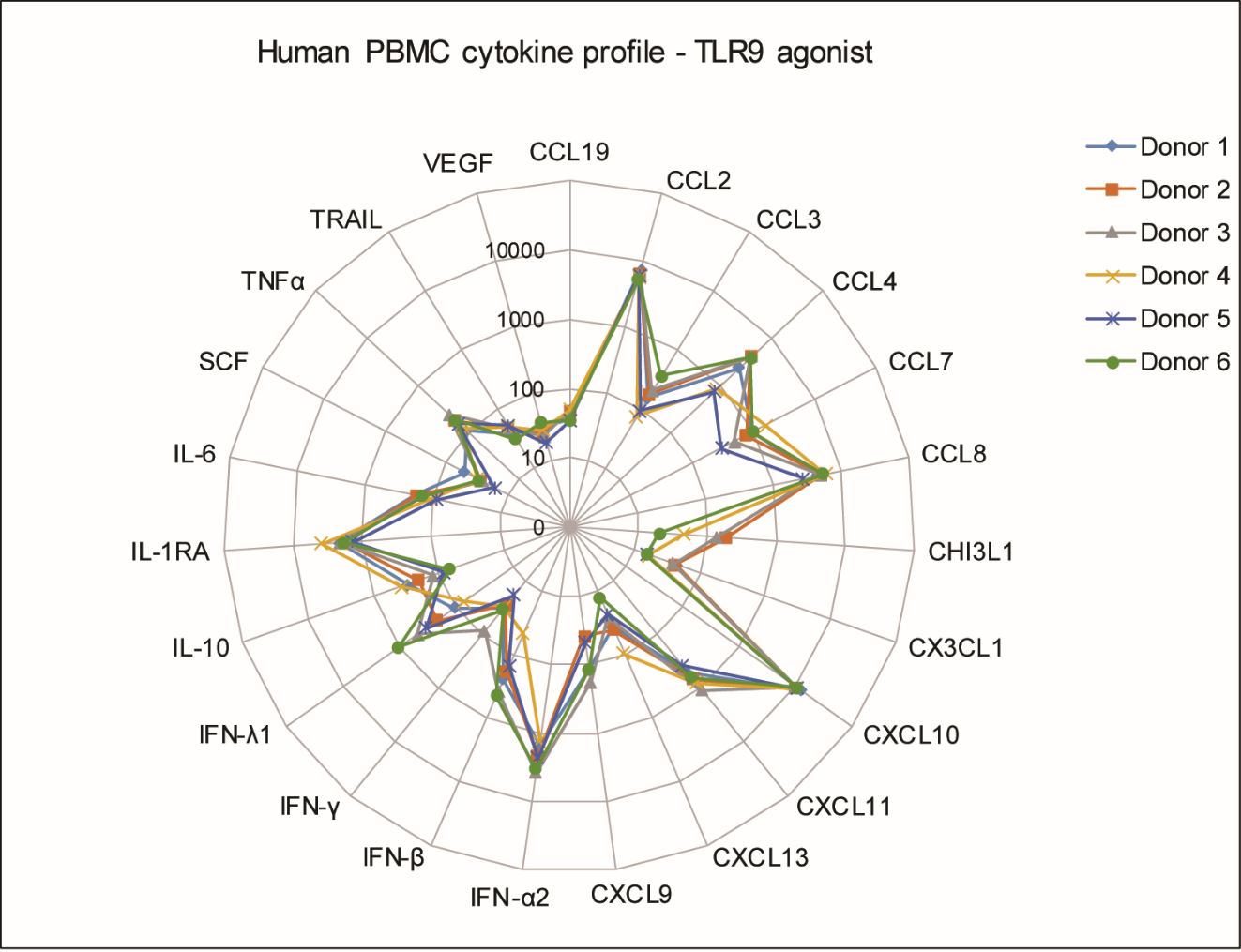


**SI Figure 10: Cytokine profiles of human PBMCs from different donors in response to TLR9 agonist.** PBMCs prepared from six healthy donors were treated with 1 μM TLR9 agonist ODN2216 for 24 hours, conditioned media were collected and subjected to Luminex multiplex assays. The concentrations of individual cytokines are background subtracted and plotted on radar chart, axis unit pg/mL. Note the data set for donor 6 is the same to the corresponding data set in supplemental Figure S8.

**SI Figure 11: IFN-β blocks IL-1β release from human iPSC-derived microglia.** Human iPSC-derived microglia cells were pre-treated with IFN-β at concentrations ranged from 50 ng/mL to 0.000069 ng/mL for 14 hours, followed by 10 ng/mL LPS priming for 4 hours and inflammasome activation with 10 μM nigericin for 2 hours. Conditioned media from microglial culture were collected for IL-1β measurement by MSD assay. Data are mean ± SD from n=2 experiments, each with three biological replicates.


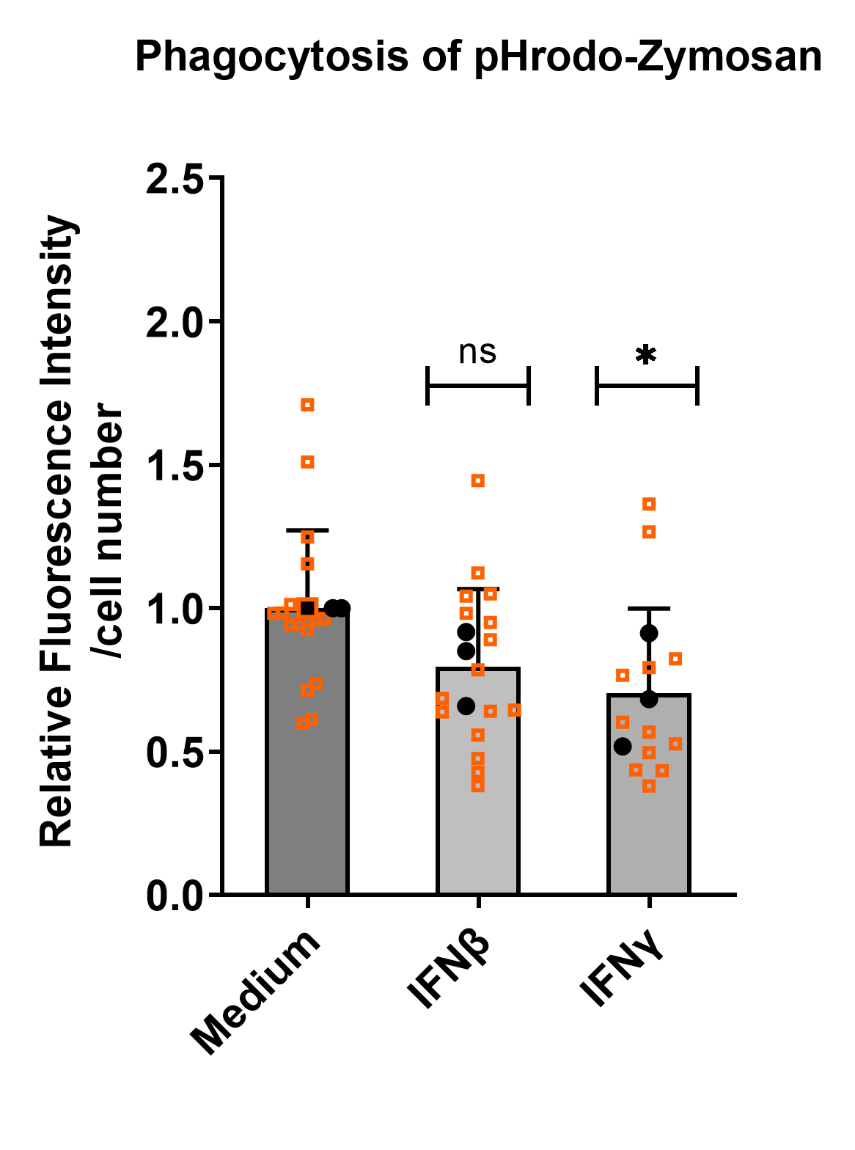


**SI Figure 12: Phagocytosis of pHrodo-labeled Zymosan in human iPSC-derived microglia treated with recombinant cytokine IFNβ or IFN-γ.** Human iPSC-derived microglial cultures were pre-treated with recombinant cytokine (IFN-β, IFN-γ) at concentration of 50 ng/ mL for ~14 hours preceding phagocytosis assay. After the pre-treatment, microglial cultures were incubated with pHrodo-labeled Zymosan (100 μg/mL) for 4 hours before live-cell scanning with the presence of CellMask™ deep red stain. Phagocytosis of pHrodo-Zymosan were quantified by dividing total fluorescence intensities of pHrodo by total cell numbers. Biological replicates from n=3 experiments (each experiment with 4-6 biological replicates) are plotted in small-sized orange squares, mean values per experiment were plotted in black dots. Mean ± SD, One-way ANOVA with Dunnett's multiple comparisons (cytokine-treated group *vs.* medium-treated group) of mean values from independent experiments. ∗p < 0.05, ns (not significant).

**
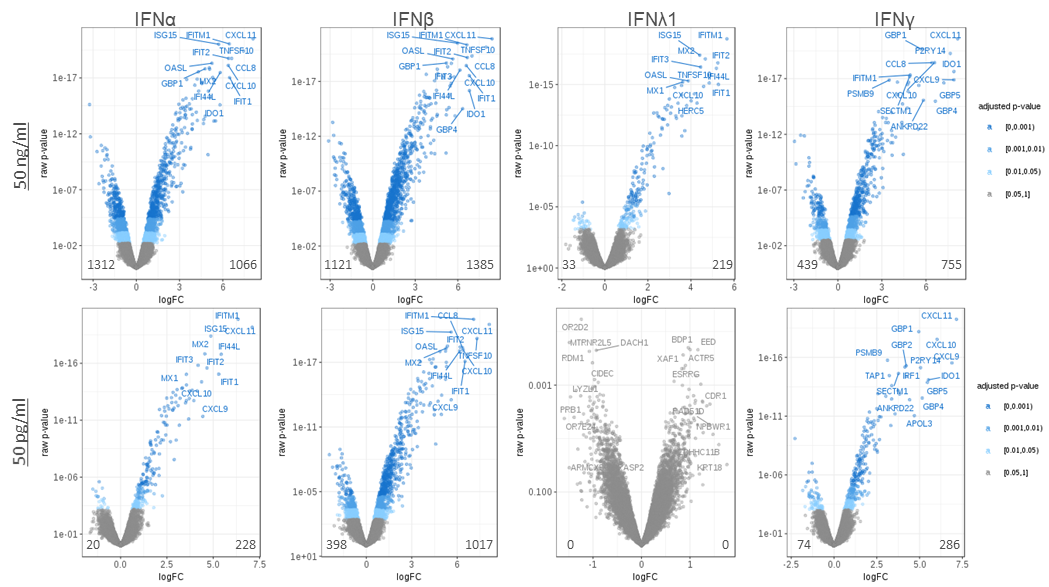
**

**SI Figure 13: Volcano plots representing differential expression induced by treatment of IFNs in human iPSC-derived microglia.** Transcriptional effects in human iPSC-derived microglia treated with 50 ng/mL or 50 pg/mL IFN-α, β, λ and γ (~14 hours) were plotted to illustrate the statistical significance (P value) versus magnitude of log_2_ fold change of individual genes. The numbers of significantly upregulated and downregulated genes are indicated at lower right and left corner, respectively.

**SI Figure 14: Principal component analysis (PCA) plot of microarray data from IFNs- treated microglia focusing on genes involved in phagocytosis pathways.** Microarray data from human iPSC-derived microglia treated with 50 ng/mL (high) or 50 pg/mL (low) IFN-α, β, λ and γ (~14 hours) were subjected to principal component analysis focusing on genes involved in phagocytosis pathways as defined by Gene Ontology Biological Processes. The top genes that mostly drive the transcriptional variations among all samples are indicated.

**SI Figure 15: TLR9-signaling induced cytokines increase expression of AXL and RUBICON.** Human iPSC-derived microglia cells were treated with condition media from human PBMCs with or without TLR9 activation. RNA extraction from the microglia cells were subjected to RT-qPCR analysis using TaqMan assays targeting AXL and RUBICON. Reference genes ACTB and TBP were used for normalization. PBMC samples were prepared from two healthy donors. Data are mean ± SD from two biological replicates.

**Reference list**

1. Hartl D, May P, Gu W, Mayhaus M, Pichler S, Spaniol C *et al.* A rare loss-of-function variant of ADAM17 is associated with late-onset familial Alzheimer disease. *Mol Psychiatry* 2020; **25**(3)**:** 629-639.

2. Auton A, Brooks LD, Durbin RM, Garrison EP, Kang HM, Korbel JO *et al.* A global reference for human genetic variation. *Nature* 2015; **526**(7571)**:** 68-74.

3. Karczewski KJ, Francioli LC, Tiao G, Cummings BB, Alfoldi J, Wang Q *et al.* The mutational constraint spectrum quantified from variation in 141,456 humans. *Nature* 2020; **581**(7809)**:** 434-443.

4. Cacace R, Heeman B, Van Mossevelde S, De Roeck A, Hoogmartens J, De Rijk P *et al.* Loss of DPP6 in neurodegenerative dementia: a genetic player in the dysfunction of neuronal excitability. *Acta Neuropathol* 2019.

5. Schwabenland M, Bruck W, Priller J, Stadelmann C, Lassmann H, Prinz M. Analyzing microglial phenotypes across neuropathologies: a practical guide. *Acta Neuropathol* 2021; **142**(6)**:** 923-936.

6. Cacace R, Heeman B, Van Mossevelde S, De Roeck A, Hoogmartens J, De Rijk P *et al.* Loss of DPP6 in neurodegenerative dementia: a genetic player in the dysfunction of neuronal excitability. *Acta Neuropathologica* 2019; **137**(6)**:** 901-918.

7. Kleinberger G, Wils H, Ponsaerts P, Joris G, Timmermans JP, Van Broeckhoven C *et al.* Increased caspase activation and decreased TDP-43 solubility in progranulin knockout cortical cultures. *J Neurochem* 2010; **115**(3)**:** 735-747.

8. Haenseler W, Sansom SN, Buchrieser J, Newey SE, Moore CS, Nicholls FJ *et al.* A Highly Efficient Human Pluripotent Stem Cell Microglia Model Displays a Neuronal-Co-culture-Specific Expression Profile and Inflammatory Response. *Stem Cell Reports* 2017; **8**(6)**:** 1727-1742.

9. Guarda G, Braun M, Staehli F, Tardivel A, Mattmann C, Forster I *et al.* Type I interferon inhibits interleukin-1 production and inflammasome activation. *Immunity* 2011; **34**(2)**:** 213-223.

10. Gentleman RC, Carey VJ, Bates DM, Bolstad B, Dettling M, Dudoit S *et al.* Bioconductor: open software development for computational biology and bioinformatics. *Genome Biol* 2004; **5**(10)**:** R80.

11. Irizarry RA, Hobbs B, Collin F, Beazer-Barclay YD, Antonellis KJ, Scherf U *et al.* Exploration, normalization, and summaries of high density oligonucleotide array probe level data. *Biostatistics* 2003; **4**(2)**:** 249-264.

12. Dai M, Wang P, Boyd AD, Kostov G, Athey B, Jones EG *et al.* Evolving gene/transcript definitions significantly alter the interpretation of GeneChip data. *Nucleic Acids Res* 2005; **33**(20)**:** e175.
